# LTD is involved in the formation and maintenance of rat hippocampal CA1 place-cell fields

**DOI:** 10.1038/s41467-020-20317-7

**Published:** 2021-01-04

**Authors:** Donovan M. Ashby, Stan B. Floresco, Anthony G. Phillips, Alexander McGirr, Jeremy K. Seamans, Yu Tian Wang

**Affiliations:** 1 Hotchkiss Brain Institute, University of Calgary, 3330 Hospital Drive NW, Calgary, T2N 4N1 AB Canada; 2 Djavad Mowafaghian Centre for Brain Health, University of British Columbia, 2215 Wesbrook Mall, Vancouver, V6T 1Z7 BC Canada; 3 Department of Psychology, University of British Columbia, 2136 West Mall, Vancouver, V6T 1Z4 BC Canada; 4 Department of Psychiatry, University of British Columbia, 2255 Wesbrook Mall, Vancouver, V6T 2A1 BC Canada; 5 Department of Psychiatry, University of Calgary, 3330 Hospital Dr NW, Calgary, T2N 4N1 AB Canada; 6 Department of Medicine, University of British Columbia, 2775 Laurel Street, 10th Floor, Vancouver, V5Z 1M9 BC Canada

**Keywords:** Hippocampus, Spatial memory, Long-term depression

## Abstract

Hippocampal synaptic plasticity includes both long-term potentiation (LTP) and long-term depression (LTD) of synaptic strength, and has been implicated in shaping place field representations that form upon initial exposure to a novel environment. However, direct evidence causally linking either LTP or LTD to place fields remains limited. Here, we show that hippocampal LTD regulates the acute formation and maintenance of place fields using electrophysiology and blocking specifically LTD in freely-moving rats. We also show that exploration of a novel environment produces a widespread and pathway specific de novo synaptic depression in the dorsal hippocampus. Furthermore, disruption of this pathway-specific synaptic depression alters both the dynamics of place field formation and the stability of the newly formed place fields, affecting spatial memory in rats. These results suggest that activity-dependent synaptic depression is required for the acquisition and maintenance of novel spatial information.

## Abstract (web summary)

LTP and LTD are involved in shaping hippocampal place field representations. Here, the authors show that de novo pathway-specific hippocampal LTD changes dynamics and stability of newly formed place fields, regulating acquisition and maintenance of novel spatial information in adult rats.

## Introduction

The exploration and encoding of novel environments is a fundamental learning process that occurs on a relatively short time scale and serves as a useful model for studying how the hippocampus encodes and represents complex, arbitrary associations required for episodic memory. Hippocampal synaptic plasticity, particularly long-term potentiation (LTP) and long-term depression (LTD), is thought to be an important subcellular substrate for spatial learning and memory^1,2^. Importantly, novelty exploration facilitates the induction of LTD^3–5^ and LTP^4,6^, the two principal forms of synaptic plasticity in area CA1 of the hippocampus^7–10^.

Pyramidal neurons in areas CA1 and CA3 of the hippocampus consistently show place modulated firing patterns called “place fields”^11^, and individual “place cells” are considered prime substrates for hippocampal encoding of spatial environments during spatial learning^12,13^. Several lines of evidence indicate that stable place field firing location is necessary for this mnemonic role of the hippocampus. Place field locations are usually consistent across exposures to a consistent environment^14–17^, and spatial navigation deficits are associated with instability of place field locations in ambiguous environments^11,18,19^.

Importantly, interventions that affect synaptic plasticity have been shown also to affect the location stability of place cells^2,20^. However, these previous demonstrations of a role for synaptic plasticity in place field maintenance used interventions that generally block both LTP and LTD, such as NMDA receptor blockade^20^, reduced PKA expression^21^, or protein synthesis inhibition^22^. Therefore, the specific role of LTP and LTD in regulating place cell encoding dynamics and the subsequent consolidation of their stable place fields remain to be specified.

Prior attempts to specify the effect of synaptic plasticity inhibition on place field formation failed to find any acute effect on place field formation dynamics. Place fields are not immediately present in a novel environment, but develop over a time-course of minutes concurrent with exploration of these environments^23–26^. Therefore it is important to investigate whether changes in synaptic plasticity occur on the same time-scale during exposure to a novel environment and whether plasticity blockade at this time-scale affects dynamics of place field formation.

**Fig. 1:**
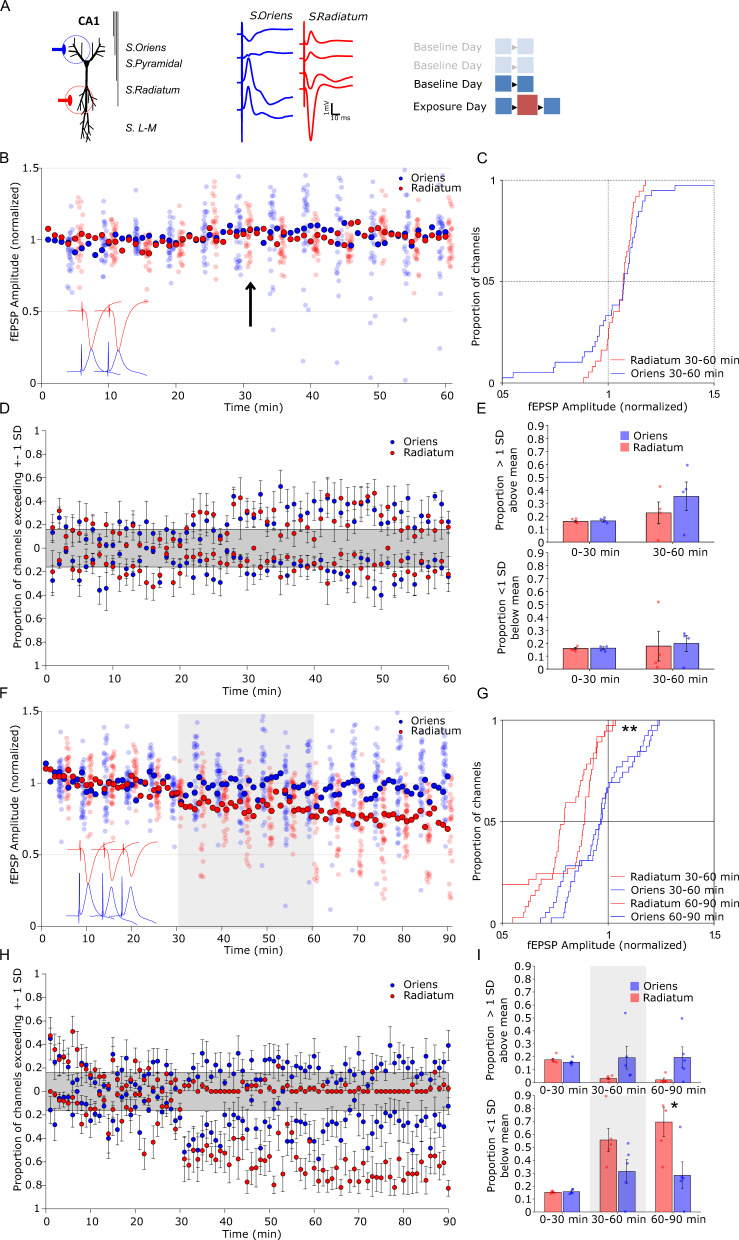
Exposure to a novel environment elicits a decrease in fEPSP specific to stratum radiatum stimulation. **A** Each tract was composed of four electrodes that spanned the laminar extent of CA1. Two stimulation electrodes were positioned to activate schaffer collateral inputs to either basal or apical CA1 dendrites in the stratum oriens or stratum radiatum respectively. Stratum oriens stimulation evoked a negative going potential in superficial electrodes and a positive going potential in deep electrodes, whereas stratum radiatum stimulation did the opposite. Three baseline days preceded the novel environment exposure day, wherein the familiar environment was presented before and after the novel environment. **B** On the final baseline day evoked field potentials are stable. Two 30-min recordings in a familiar recording chamber were separated by a brief handling episode (arrow). Solid marker indicates the cross-channel average, while each channel is plotted every 5 stimulations and offset for clarity. Hippocampal CA1 field potentials evoked from stimulation of the oriens or radiatum pathway showed no significant change across time. **C** Cumulative distribution of evoked field potential change in the second 30-min epoch relative to the first (blue, oriens, *n* = 38 channels; red, radiatum, *n* = 38 channels). **D** Per-animal proportion of channels exceeding 1 standard deviation of the channel’s baseline variance at each stimulation. Shaded area represents the proportion of channels expected to be above or below 1 standard deviation based on a normal distribution (*n* = 5 rats). **E** Proportion of channels exceeding 1 standard deviation above (top graph) or below (bottom graph) baseline, in 30-min bins. No significant change in distribution was observed on the baseline day in either channel (*n* = 5 rats). **F** On the exposure day, A 30-min baseline recording was followed by 30 min in a novel environment (gray shading), followed again by 30 min in the familiar recording chamber. Solid marker indicates the cross-channel average, while each channel is plotted every 5 stimulations and offset for clarity. **G** Cumulative distribution of amplitude change relative to baseline period during novelty (solid) and after returning to the familiar chamber (dashed) showed a widespread decrease in radiatum evoked field potential distributions relative to the oriens pathway (blue, oriens, *n* = 38 channels;red, radiatum, *n* = 38 channels). **H** Per-animal proportion of channels exceeding 1 standard deviation of the channel’s baseline variance at each stimulation (*n* = 5). **I** Proportion of channels averaged in each epoch indicate a specific increase in the proportion of channels lower than 1 standard deviation from baseline in the radiatum channel after exposure to the novel environment (*n* = 5). *p < 0.05, **p < 0.01 follow-up *t*-test (two-way) comparison between treatment group for epochs after significant group by epoch ANOVA interaction, and k-s test for distribution. Error bars represent ± SEM.

Here, we use multi-electrode electrophysiology to examine whether novelty exploration results in de novo changes in synaptic strength across multiple recording sites in stratum radiatum inputs to apical dendrites of hippocampal CA1 neurons, and if so, whether they are broadly observed across all sites, as reported for hippocampal-dependent aversive learning^27^. Historically, behaviorally driven changes in the strength of evoked field responses have been confounded by changes in body temperature and behavioral state^28,29^. To control for these and other unknown variables, we adopted an internal control by using a second stimulating electrode to activate dendritic inputs in the striatum oriens (Fig. 1A). These basal dendritic synapses also exhibit LTP and LTD^30^, but are not known to be affected by novelty or stress. We then observe the formation of place-cell fields in a novel environment and their stability across days in the presence of a specific pharmacological inhibitor of LTD expression to determine whether LTD participates in stable place representation in the hippocampus.

## Results

### Novelty triggers pathway-specific synaptic depression

Stimulation at both stratum radiatum and stratum oriens evoked canonical post-synaptic field potentials (fEPSPs, Fig. 1A) that were maintained for 1-h recording sessions across at least 3 days. Fig. 1B illustrates stable average responses in the final baseline session, after brief handling after 30 min to simulate environmental transfer. Individual channel responses were plotted every 5 stimulations for clarity. No change in the distribution of responses between channels was observed in the baseline session (Fig. 1C; Supplemental Table 1). On the exposure day rat fEPSPs were evoked once per minute (0.16 Hz) through a 30-min baseline period in the familiar recording chamber, followed by a subsequent 30-min period in a novel environment and a final 30-min returned to the familiar environment (Fig. 1F). The distribution of baseline normalized radiatum responses was significantly changed relative to oriens evoked responses during and after novel environment exploration (Fig. 1G; ks_30–60_ = 0.37, *p* < 0.01, ks_60–90_ = 0.47, *p* < 0.001), with nearly every channel showing a small decrease in amplitude over the duration of the recording session. While the distribution of responses were significantly shifted in the radiatum group relative to oriens, these results aggregated multiple recording locations from multiple animals and encompassed considerable variation in fEPSP change. Therefore we sought to determine whether a change in synaptic strength was evident on a per-animal basis. Previous research using multiple channels has used a 1 standard deviation (SD) threshold to examine deviations from baseline that do not depend on the absolute magnitude of the deviation or a homogenous shift on all channels^27^. Calculating an average proportion of channels above or below 1 SD on a per animal basis demonstrated that a specific decrease in a subset of radiatum evoked fEPSPs was a consistent feature across animals relative to oriens evoked fEPSPs after exposure to the novel environment (Fig. 1H, I; *F*_Epoch_(2,8) = 16.17, *p* < 0.001, *F*_Group_(1,8) = 5.81, *p* = 0.04, *F*_Epoch*Group_(2,8) = 5.49, *p* = 0.02 interaction, follow-up pairwise comparisons oriens vs radiatum *t*_0–30_(8) = 0.55, *p* = 0.59, *t*_30–60_(8) = 1.89, *p* = 0.09, *t*_60–90_(8) = 2.67, *p* = 0.02). No such alteration was observed in the baseline recording session on the prior day (Fig. 1D, E; Supplementary Table 1). These results demonstrate that the novel environment induced a de novo and pathway-specific reduction in field potential magnitude at apical inputs to hippocampal CA1 neurons.

### fEPSP depression is mediated by AMPA receptor endocytosis

**Fig. 2:**
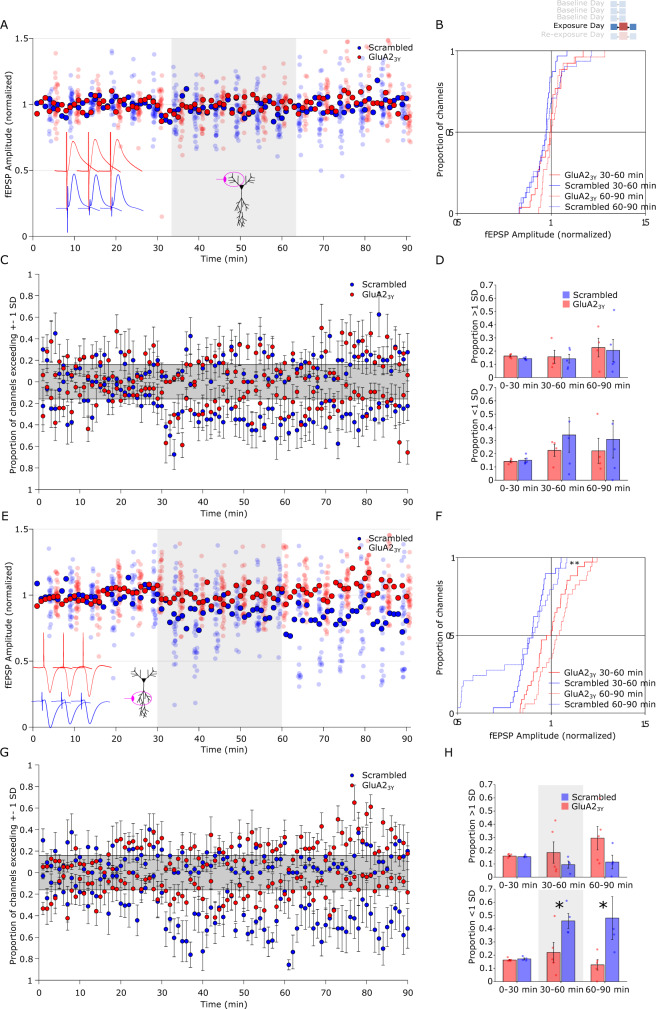
Novelty exposure induced fEPSP reduction is blocked by AMPA-receptor endocytosis inhibition. tat-GluA2_3Y_ peptide or a Scrambled control (2.25 μmol/kg in saline, IV) was delivered 30 min prior to recording on the exposure day. **A** Stratum oriens stimulation evoked fEPSPs in GluA2_3Y_ (red) or Scrambled (blue) treated rats. Solid marker indicates the cross-channel average, while each channel is plotted every 5 stimulations and offset for clarity. **B** Cumulative distribution of oriens-evoked fEPSPs showed no difference between treatment groups during or after novelty exposure (red, GluA2_3Y_ *n* = 26 channels; blue, Scrambled, *n* = 31 channels). **C** Per-animal proportion of channels exceeding 1 standard deviation of the channel’s baseline variance at each stimulation. Shaded area represents the proportion of channels expected to be above or below 1 standard deviation based on a normal distribution. (*n* = 4 GluA2_3Y_, *n* = 5 Scrambled). **D** Proportion of channels exceeding 1 standard deviation above (top graph) or below (bottom graph) baseline in each epoch. No significant change in distribution was observed in either treatment group during or after novelty exposure treatment (*n* = 4 GluA2_3Y_, *n* = 5 Scrambled). **E** Stratum radiatum evoked fEPSPs in GluA2_3Y_ (red) and Scrambled (blue) treated rats. **F** Cumulative distribution of radiatum-evoked fEPSPs showed a widespread decrease during novelty exposure (solid) and on return to the familiar chamber (dashed) that was blocked by GluA2_3Y_ treatment (red, *n* = 34 channels; blue, Scrambled, *n* = 29 channels). **G** Per-animal proportion of channels exceeding 1 standard deviation of the channel’s baseline variance at each stimulation (*n* = 5 GluA2_3Y_, *n* = 4 Scrambled). **H** Proportion of channels averaged in each epoch indicate a specific increase in the proportion of channels lower than 1 standard deviation from baseline in the radiatum channel during after exposure to the novel environment that was blocked by GluA2_3Y_ treatment (*n* = 5 GluA2_3Y_, *n* = 4 Scrambled). *p < 0.05, **p < 0.01 follow-up *t*-test (two-tail) comparison between group for epochs after significant group by epoch ANOVA interaction, and ks-test for distribution. Error bars represent  ± SEM.

To test whether the observed de novo fEPSP depression was mediated by a facilitation of endocytosis of postsynaptic AMPA receptors, a well-characterized mechanisms of LTD induced by electrical stimulation^31,32^, the GluA2_3Y_ (previously known as GluR2_3Y_) interference peptide or a Scrambled control peptide was administered IV prior to the recording session on the novelty exposure day after baseline recording days (Supplementary Fig. 1) in a separate cohort of animals. Oriens evoked potentials in response to novelty exploration showed no alteration in the distribution of responses between GluA2_3Y_ and Scrambled treatments (Fig. 2A, B; Supplementary Table 1). In contrast, the de novo shift in fEPSP responses observed at radiatum sites was specifically prevented by GluA2_3Y_ relative to Scrambled control after novel environment exploration (Fig. 2E, F; ks_30–60_ = 0.27, *p* = 0.18, ks_60–90_ = 0.49, *p* < 0.001). This distribution change was reflected in the per-animal proportion of channels below 1 SD of baseline (Fig. 2G, H; *F*_Epoch_(2,7) = 3.21, *p* = 0.07, *F*_Group_(1,7) = 8.11, *p* = 0.02, *F*_Epoch*Group_(2,7) = 3.60, *p* = 0.05; follow-up pairwise comparisons Scrambled vs GluA2_3Y_ *t*_0–30_(7) = 1.08, *p* = 0.32, *t*_30–60_(7) = 2.41, *p* = 0.047, *t*_60–90_(7) = 2.38, *p* = 0.049). No significant effect of drug treatment was observed in the oriens pathway on the exposure day (Fig. 2C, D; Supplementary Table 1). On the following day, rats were re-exposed to the same novel environment without drug infusions. In radiatum pathways, a transient alteration in the distribution fEPSP magnitude was observed with opposing directionalities between the novel environment and the post-novelty period (Supplementary Fig. 2, ks_30–__60_ = 0.58, *p* < 0.001, ks_60–90_ = 0.32, *p* = 0.06) with no alteration in distribution in the oriens pathways (Supplementary Table 1). This was reflected in a marginal non-significant increase in the per-animal proportion of channels 1 SD higher than baseline in the Scrambled treated group relative to GluA2_3Y_ (Supplementary Fig. 2; *F*_Epoch_(2,7) = 4.20, *p* = 0.04, *F*_Group_(1,7) = 0.59, *p* = 0.47, *F*_Epoch*Group_(2,7) = 3.74, *p* = 0.05). No significant effect of drug treatment was observed in the oriens pathway on the re-exposure day (Supplementary Fig. 2; Supplementary Table 1). Together these results show that novel environment exposure produces a de novo reduction in fEPSP magnitude in the apical inputs to CA1 that is mediated by activity-dependent AMPA receptor endocytosis, a well-characterized mechanism for LTD expression^31,32^. The novel environment elevated locomotion relative to the familiar environment in both GluA2_3Y_ and Scrambled peptide treated rats (Supplementary Fig. 7), with no difference between drug treatment groups. As locomotor activity itself could conceivably induce pathway-specific plasticity^33,34^, we compared activity levels during the first exploration of the novel environment to the re-exploration on the following day. Though fEPSP reductions were only observed on the first exploration, activity levels were comparable between exploration and the first re-exploration, suggesting that novelty itself is a necessary driver of the plasticity effect. Although some previous studies have reported fEPSP decreases triggered by exposure to novel environments^3,27^, it was unclear whether this effect was pathway-specific or mediated by synaptic plasticity mechanisms. We show here that a widespread synaptic change in response to environmental novelty is pathway-specific fEPSP depression mediated by AMPA receptor endocytosis. Our results suggest that while this reduction is widespread it is also heterogenous, with some channels undergoing a larger reduction than others.

### New place field maintenance requires synaptic depression

**Fig. 3:**
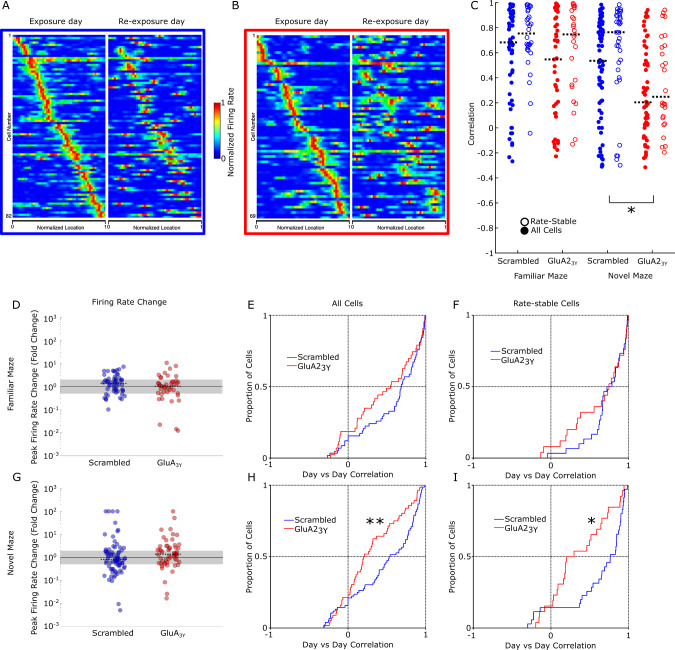
Blocking AMPA receptor endocytosis impairs place field stability between days in a novel environment. **A** All matched cells recorded in the linear novel environment on the exposure day (left) and re-exposure day (right) from Scrambled peptide treated rats, sorted by firing location on the exposure day show a preserved pattern on the re-exposure day. **B** All matched cells recorded in the linear novel environment on the exposure day (left) and re-exposure day (right) from GluA2_3Y_ treated rats, sorted by firing location on the exposure day show an impaired spatial pattern on re-exposure. **C** Day over day place field correlations for all matched cells (filled; Scrambled *n* = 58 familiar, *n* = 76 novel, from *N* = 6 rats blue; GluA2_3Y_ *n* = 43 familiar, *n* = 56 novel, from *N* = 6 rats red) and rate-stable cells (open; Scrambled *n* = 30 familiar, *n* = 35 novel from *N* = 6 rats; GluA2_3Y_ *n* = 25 familiar, *n* = 26 novel from *N* = 6 rats). Black bar indicates group median correlation. **D**, **G** Firing rate changes from day to day in Scrambled and GluA2_3Y_ peptide treated rats on the familiar and novel maze. Median firing rate changes were minimal in all conditions. Gray indicates rate-stable cells, with rate changes between 0.5 and 2. **E**, **H** Day over day correlation distributions in the familiar (**D**) and novel (**G**) linear mazes, with peptide (red) and Scrambled (blue) treatment. GluA2_3Y_ treated rats had significantly reduced correlations relative to Scrambled treatment in the novel environment. **F**, **I** Day over day correlation in rate-stable cells in the familiar (**E**) and novel (**H**) linear mazes, with peptide (red) and Scrambled (blue) treatment. GluA2_3Y_ treated cells were significantly less correlated in the novel environment, even in rate-stable cells. *p < 0.05, **p < 0.01, k-s test for cumulative distribution or Mann–Whitney *U* follow-up pairwise comparisons (two-tailed). Error bars represent ± SEM.

The widespread de novo fEPSP reduction we observed suggests that rather than being a direct encoding mechanism for the new environment, this synaptic change may participate in a gain-control type function^35^. Accordingly, de novo fEPSP reduction may act in concert with other plasticity changes such as LTP in other hippocampal pathways to encode the new environment. To test this conjecture directly, we next investigated whether blocking AMPA receptor endocytosis during exposure to a novel environment would affect the hippocampal encoding of that environment. Place field formation occurs in a novel environment, and these newly formed place fields are specifically affected by NMDA receptor blockade, CAMKII deletion, and protein synthesis inhibition^20,21,36–38^. While these interventions likely block both synaptic LTP and LTD, LTP is thought to be the mediator of these effects^39,40^ and the specific role of blocking LTD has not been investigated. Measurement of place field formation dynamics is facilitated in a single dimensional maze where laps are completed more rapidly and with greater consistency. Accordingly, we employed a linear maze composed of reconfigurable sections that permitted multiple different environments (Supplementary Fig. 3) to examine the effect of LTD blockade. A total of 458 cells from seven rats were recorded during exploration of linear environments. Place field stability across days was achieved by the use of waveform characteristics (Supplementary Fig. 4) to identify matching cells^41,42^, and field stability was measured as a correlation between the firing maps produced in two different sessions from cells with matching waveform characteristics. As place fields can remap exclusively via either a rate change (rate remapping) or by changing field location (global remapping), either of which may account for reduced correlations, we additionally assessed place field stability in the subset of cells with stable firing rates, defined as a peak rate that changed between days by less than a factor of 2 (Fig. 3D, G). Place field firing was recorded in a highly familiar linear configuration on a baseline day. The subsequent day, GluA2_3Y_ or Scrambled control peptide was administered IV, and a novel maze configuration was presented after an initial exploration of the familiar environment. Following exposure to the novel configuration, rats were returned to the familiar environment. The same protocol was employed the following day in the absence of drug administration. Rats administered Scrambled peptide prior to novelty exposure showed primarily correlated place fields between days on the novel maze, both overall and in the subset of rate-stable cells (Fig. 3C, Supplementary Table 1). In contrast, following administration of the interference peptide GluA2_3Y_, the novel environment place fields displayed significantly less conservation between days when compared as a distribution to Scrambled fields both overall (Fig. 3B, H; ks = 0.31, *p* = 0.003) and in the rate-stable subset of cells (Fig. 3I; ks = 0.40, *p* = 0.013). GluA2_3Y_ peptide treatment did not impair place field correlation distributions in the familiar environment overall (Fig. 3E) or in rate-stable cells (Fig. 3F). As correlations were skewed towards 1 in most groups, a non-parametric one-way ANOVA (Kruskal–Wallis) on four groups (Familiar/Novel Maze, Scrambled/GluA2_3Y_ treatment) was conducted on overall correlations and rate-stable correlations to investigate whether average place field correlations were affected by GluA2_3Y_ treatment. A significant difference between groups (overall, *χ*^2^(3) = 16.07, *p* = 0.0011; rate-stable, *χ*^2^(3) = 14.02, *p* = 0.0029) with follow-up targeted pairwise comparisons (Mann–Whitney *U*) showed that GluA2_3Y_ treatment reduced place field stability specifically in the novel environment (Fig. 3C, overall, *U*_Novel_ = 2.59, *p* = 0.0096; rate-stable, *U*_Novel_ = 2.63, *p* = 0.0085). These data suggest that reduced correlations are neither a generalized consequence of GluA2_3Y_ treatment nor specifically due to changes in firing rates. Therefore, we conclude that disruption of an LTD expression mechanism by GluA2_3Y_ induced aberrant global remapping of a novel environment. Similar results were obtained with exploration of a novel two dimensional square environment that matched the geometry used to observe LTD of evoked field potentials (Supplementary Fig. 5).

### AMPA receptor endocytosis inhibition alters field formation

Stable place fields form and consolidate over the first several minutes of exploration in a novel environment^23,25,26,43^. The time scale of these dynamics are consistent with the de novo fEPSP reduction we observed. Additionally, experiments that record field firing in mice exploring virtual environments^44^ report similar dynamics^45,46^ and implicate dendritic plateau potentials and other nonlinearities in the rapid recruitment of new place fields^47^. Importantly, these reports support the idea that an active synaptic plasticity process is required despite relatively short time spans, as the subthreshold voltages recorded before, during, and after recruitment change dramatically^48^. We hypothesized that AMPA receptor endocytosis inhibition may interfere with the speed of field recruitment or the early stability of these fields by raising the noise level of synaptic inputs to CA1.

**Fig. 4:**
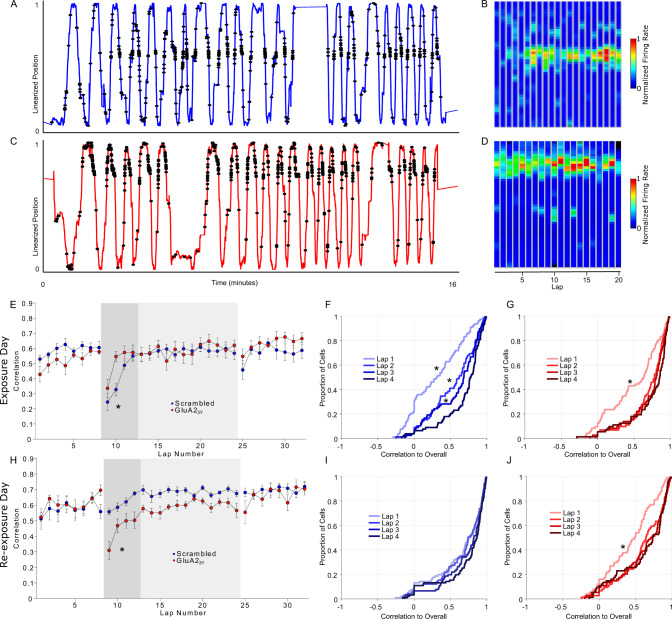
Blocking AMPA receptor endocytosis acutely alters place field formation dynamics. **A**–**D** Linearized position and spike locations for one example cell from a Scrambled treated rat (**A**) and GluA2_3Y_ treated rat (**C**) and the lap-by-lap firing field (**B**, **D**) upon first exposure to a novel environment. Faster field formation was apparent in GluA2_3Y_ treated rats. **E** On the exposure day rats were administered GluA2_3Y_ or Scrambled peptide (2.25 μmol/kg IV) prior to a baseline exploration of the familiar configuration, followed by exploration of a novel configuration (gray shading). Place fields were highly correlated and similar between groups in the familiar configuration (*n* = 76 Scrambled in *N* = 6 rats, *n* = 60 GluA2_3Y_ in *N* = 6 rats), but were lower in the first several laps of a novel configuration (dark gray shading). Cells from GluA2_3Y_ treated rats were highly correlated by the second lap, relative to Scrambled peptide treated rats (Scrambled *n* = 76 baseline, *n* = 78 novel from *N* = 6 rats; GluA2_3Y_ *n* = 60 baseline, *n* = 72 novel from *N* = 6 rats). **F** The cumulative distribution of correlations on the first four laps developed progressively over four trials in Scrambled treated rats. **G** Highly correlated fields were established after a single lap in GluA2_3Y_ peptide treated rats. **H** Rats previously administered GluA2_3Y_ peptide (red) or Scrambled peptide (blue) were re-exposed to the novel environment (gray shading) after baseline exploration of the familiar environment. Lap by lap correlations were high in the familiar configuration in both groups, however only rats previously treated with Scrambled peptide showed highly correlated fields in the early laps on the previously novel environment (Scrambled *n* = 80 baseline, *n* = 91 novel from *N* = 6 rats; GluA2_3Y_ *n* = 67 baseline, *n* = 74 novel from *N* = 6 rats). **I** Cumulative distribution of correlations over the first four laps showed highly correlated field on re-exposure in Scrambled peptide treated rats. **J** Re-exposure in GluA2_3Y_ treated rats showed evidence of consolidation over early laps. In contrast, correlations developed over several laps in rats treated with GluA2_3Y_ peptide, reminiscent of the initial exposure to the environment in control rats. * indicates *p* < 0.05 follow-up *t*-test (two-way) comparison after significant lap by group ANOVA interaction, and *p* < 0.05 ks-test vs lap 4 distribution. Error bars represent ± SEM.

In our experimental design, rats traversed the entire maze more than once a minute, and therefore the linearized maze design permitted an analysis of firing field stability within a session (Supplementary Fig. 3). Firing rate maps were computed for each individual lap, and a correlation matrix was calculated for each cell, with the median correlation of each lap to each other lap taken as a similarity index. As individual rats completed varying numbers of laps within the fixed time recording session, the first eight laps were averaged for familiar configuration sessions (8-min sessions) and the first 16 laps were averaged for the novel configuration sessions (16-min sessions). Lap to lap similarly was uniformly high in the familiar environment (Supplementary Fig. 6). Consistent with prior reports, in the novel environment the correlations between average place cell firing maps increased over the first several laps, with significantly lower correlations until the fourth lap in control rats (Fig. 4A, B, E).

In contrast to our hypothesis, recruitment speed and field stability were not impaired by GluA2_3Y_ peptide treatment, however an alteration in dynamics was observed, with highly correlated place fields established by the second lap (Fig. 4C–E). A comparison of the distribution of correlations specifically on the first four laps demonstrated a rapid, single lap shift to a high correlation distribution in GluA2_3Y_ treated rats (Fig. 4G; ks-test vs lap 4: ks_Lap1_ = 0.37, *p* < 0.001, all other laps *p* > 0.05), whereas a cumulative shift over four laps was evident in control rats (Fig. 4F; ks-test vs lap 4: ks_Lap1_ = 0.52, *p* < 0.001; ks_Lap2_ = 0.29, *p* = 0.0017; ks_Lap3_ = 0.22, *p* = 0.042). These unexpected results indicate that AMPA receptor endocytosis blockade does not acutely diminish the lap to lap stability of place fields. Instead, this intervention appeared to accelerate the formation of a stable place field during novelty exposure. On a subsequent re-exposure day, place fields from Scrambled peptide treated rats were stable from the first lap of the re-exposed novel environment (Fig. 4H, I; Supplementary Table 1). In contrast, place fields were initially unstable in rats treated with GluA2_3Y_ peptide (Fig. 4H, J,*p* < 0.05 lap 1 vs 4), reminiscent of place field dynamics in a novel environment. Accordingly, AMPA receptor endocytosis inhibition appears to exert two unique and opposing effects on the formation of new place fields in a novel environment. Specifically, following treatment with GluA2_3Y_ peptide, stable place fields form more rapidly within the session as compared with controls, however place fields are not maintained across days as is typically observed.

### Synaptic depression is required for new contextual learning

**Fig. 5:**
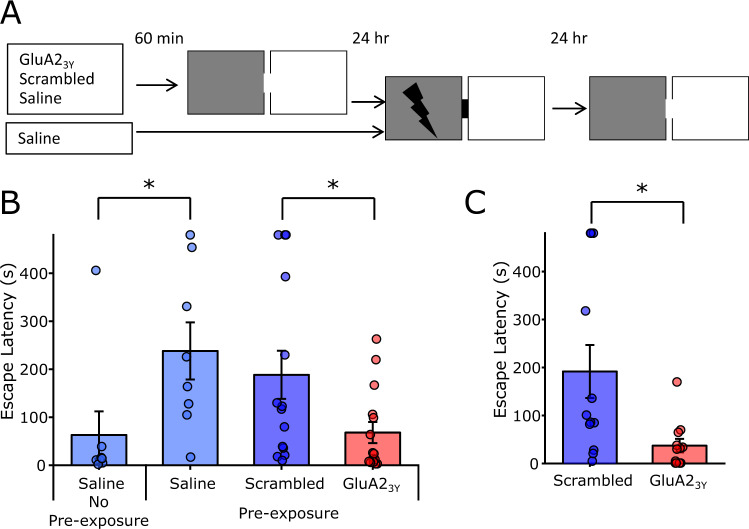
Blocking AMPA receptor endocytosis impairs contextual pre-exposure facilitation of inhibitory avoidance. **A** 60-min prior to contextual pre-exposure (8-min free exploration of both chambers), rats were administered GluA2_3Y_ peptide (*n* = 15;2.25 μmol/kg IV), Scrambled control (*n* = 14), or saline vehicle (*n* = 8). A fourth group was only administered saline without contextual pre-exposure (*n* = 8). 24 h later rats were conditioned by direct placement into the dark compartment followed by immediate shock (2 × 0.4 mA, 0.5 s shock for IV experiment, 2 × 0.55 mA, 0.5 s shock for IC experiment) and removal. 24 h after training, rats were place in the light compartment and escape latency to the dark compartment was measured. **B** Saline treated rats with no contextual pre-exposure (*n* = 8) show poor memory relative to pre-exposed rats (*n* = 8). Systemic GluA2_3Y_ (*n* = 15) administered during pre-exposure impaired inhibitory avoidance conditioning relative to a Scrambled control (*n* = 14). **C** Local microinjection of GluA2_3Y_ (*n* = 12; 45 pmol/μl, 2.5 μl/hemisphere IC) in the dorsal hippocampus similarly impaired inhibitory avoidance conditioning relative to Scrambled control (*n* = 12). * Indicates *p* < 0.05 pairwise comparison. Error bars represent ± SEM.

These effects of GluA2_3Y_ on place field stability may suggest that AMPA receptor endocytosis inhibition interferes with hippocampal-dependent memory such as contextual conditioning. However our previous research challenges this assertion, as LTD blockade by either GluA2_3Y_ peptide or GluN2B subtype NMDA receptor antagonism did not impair contextual fear conditioning^49^. Contextual fear conditioning is greatly affected by prior habituation to the training environment context^50^, and NMDA receptor antagonism in the hippocampus is less effective in blocking inhibitory avoidance in rats subject to context pre-exposure^51,52^. We hypothesized that GluA2_3Y_ peptide induced place field instability would impair contextual pre-exposure facilitation and therefore examined the effect of peptide administration during contextual pre-exposure on subsequent expression of inhibitory avoidance (Fig. 5A). Rats not exposed to contextual environmental cues a day prior to context-shock training showed impaired inhibitory avoidance, crossing rapidly into the dark chamber paired with footshock relative to rats that received contextual pre-exposure, who showed pronounced inhibitory avoidance (Fig. 5B; *t*(14) = 2.27, *p* = 0.039). Pre-exposed rats treated with Scrambled peptide also showed robust inhibitory avoidance, however the latency to enter the dark compartment was markedly impaired in GluA2_3Y_ treated rats (Fig. 5B; *t*(27) = 2.25, *p* = 0.033). It is important to note that GluA2_3Y_ peptide was administered only during pre-exposure to the environmental cues, leaving AMPA receptor endocytosis unperturbed during context-shock association 24 h later. To establish a locus of action for this systemic effect, GluA2_3Y_ or Scrambled control peptide (45 pmol/μl, 2.5 μl/hemisphere) was microinjected into the dorsal hippocampus prior to contextual pre-exposure. Similar to the systemic effect, GluA2_3Y_ markedly reduced inhibitory avoidance relative to Scrambled control treated rats (Fig. 5C; *t*(22) = 2.71, *p* = 0.013). Thus, AMPA receptor endocytosis inhibition during contextual pre-exposure is sufficient to impair the subsequent context-shock association, supporting a critical role of LTD mechanisms during novel environment exposure in contextual learning.

## Discussion

The present findings illuminate several features of hippocampal learning and support a role for synaptic LTD mechanisms in the acquisition of novel spatial information in the hippocampus. Inhibition of activity-dependent AMPA receptor endocytosis with the peptide inhibitor GluA2_3Y_, a validated and specific inhibitor of LTD expression mechanisms, blocked a novelty induced decrease in evoked fEPSPs in the hippocampus, blocked facilitation of inhibitory avoidance by contextual exposure, and impaired the maintenance of place field locations on re-exposure to a novel environment. In addition, acute AMPA receptor endocytosis inhibition altered the dynamics of place field formation in a novel environment. Although we demonstrated pathway-specific reduction in fEPSP magnitude in response to novelty exploration, we blocked AMPA receptor endocytosis throughout the brain while recording field potentials and CA1 place field activity. As the behavioral effect of systemic treatment on impaired inhibitory avoidance was recapitulated with hippocampus-specific inhibition of AMPA receptor endocytosis, our evidence suggests that mechanisms supporting LTD in the hippocampus are engaged during novel environment exposure to establish stable place field formation and memory. De novo fEPSP reduction specifically in synapses to apical dendrites of CA1 neurons in response to novelty exposure in the absence of changes in synapses targeting the basal dendrites of CA1 in the stratum oriens suggest an effect independent of global alterations such as brain state or temperature changes previously implicated in behaviorally driven alterations in synaptic strength^28,34,53^. Furthermore, we provide evidence that locomotor activity is not sufficient to produce this plasticity effect, as elevated locomotion on the second exploration of the novel environment did not produced fEPSP reductions seen on the first exploration.

We failed to observe evidence for LTP, reported to be facilitated under certain novelty conditions^4,6^, notably those that involve exploration of an empty environment in which the flooring has been altered. As this environmental change is similar to the novel environment we employed, we suggest two possible explanations for these differing results. First, LTP may only be observable with an induction stimulation protocol (i.e., high frequency stimulation). Accordingly, our data would suggest that environmental exploration creates a metaplastic environment that facilitates LTP while also inducing de novo synaptic depression. Considerable evidence^54–56^ supports the conjecture that metaplastic conditions that promote LTP can inhibit LTD and vice versa. Accordingly, this explanation would suggest a more complex interplay between LTP and LTD in reaction to novelty. A second explanation may be linked to important differences between the environmental exploration paradigm employed here as compared to those employed in previous studies. As our novel environment differed completely from baseline, rather than altering discrete elements of a familiar recording chamber^4^, the direction of metaplastic change may depend on some dimension of novelty magnitude. In this case our findings are consistent with prior reports confirming that facilitation of LTD can arise as a consequence of novelty exposure^3,5^.

Our observation that information transfer via inputs to basal and apical dendrites of CA1 pyramidal cells is differentially altered by novelty has implications for understanding how place fields form. Apical and basal inputs are physiologically isolated^57,58^, however both inputs contribute to the generation of the complex spiking in CA1 cells that codes for place in a freely moving animal^59^. It is important to note that apical inputs are adjacent to direct inputs from entorhinal cortex, and functional interactions between these inputs influence spiking behavior^47^ and plasticity in both pathways^60,61^. LTD in the apical Schaffer collateral input is therefore uniquely positioned to influence the likelihood that coincident inputs from the entorhinal cortex will drive cell spiking^62^.

We report an acute effect of plasticity blockade on the formation of place fields. Although these fields formed without apparent deficit, the dynamics of their formation were subtly altered, in that stable field firing formed more rapidly than expected. The initial absence and gradual formation of place fields in a novel environment has been well established^23–26^. Our results confirm these prior observations, with stable fields appearing after three to four laps of our linear maze, (i.e., the first 2–3 min). In the absence of AMPA receptor endocytosis, stability was apparent after a single lap. The regularity of established fields implies that without synaptic depression some pre-existing structure may predominate, and we suggest that LTD serves to constrain such structure. This conjecture provides a plausible explanation for the unexpected acceleration of place field formation in rats following administration of the GluA2_3Y_ peptide.

The present results are entirely consistent with prior reports of impaired maintenance of place field location after interference with synaptic plasticity^20–22^, raising the possibility they are related to effects on LTD, either exclusively or in combination with their effect on LTP. Importantly, we show that AMPA receptor endocytosis inhibition also impairs maintenance of novel spatial learning, suggesting that the effect of GluA2_3Y_ peptide on place field maintenance translates to impaired spatial memory. Most spatial and contextual learning paradigms are preceded by habituation to the training environment, and without such habituation, subsequent learning is severely impaired^50–52,63^. Our data confirm that this contextual pre-exposure facilitation effect is dependent on LTD, which we interpret as evidence that synaptic weakening is required for non-associative contextual learning. Thus although forming an association between an established context and an aversive consequence does not require LTD^49^, the initial formation of a contextual representation is LTD-dependent.

This work adds to an expanding corpus of evidence showing diverse roles for synaptic LTD in learning and memory^64^, including extinction learning^49^, behavioral flexibility^65^, time-dependent memory loss^66,67^ and impairment of recognition learning^68,69^. In the hippocampus, LTD is required for consolidation of spatial memories mediating performance in the Morris water maze^70^, and habituation to a novel object configuration^5^. These latter effects related to novelty suggest a core role for LTD in processing non-associative, highly novel information. Furthermore, exploration of a novel environment triggers behavioral and neurochemical responses that elicit LTD in the hippocampus. While it remains to be determined how this LTD impacts ongoing information processing in the hippocampus, we show here that disruption of LTD impairs consolidation of place fields, and alters their acute formation dynamics.

## Methods

### Animals

All experiments were approved by the University of British Columbia Animal Care Committee or the University of Calgary Animal Care Committee in accordance with the policies of the Canadian Council on Animal Care. Male Sprague-Dawley rats (Charles River) weighing 300–450 g were housed in pairs on a 12 h:12 h standard light cycle, or individually for freely moving electrophysiology experiments. Rats were provided food and water access *ad libitum*.

### Drugs

The interference peptide Tat-GluA2_3Y_ (YGRKKRRQRRR-869YKEGYNVYG877) and a scrambled control Tat-Scrambled (YGRKKRRQRRR-VYKYGGYNE) were synthesized in house and dissolved in saline (2.25 µmol/kg) for intravenous bolus injection.

### Probe construction

For evoked potential recordings, a probe containing 8 bundles of 4 electrodes (25 µm tungsten, California Fine Wire) was arranged in a 4 by 2 pattern. Within each bundle, electrode tips were cut at 300 µm spacing to span the strata of CA1, and bundles were separated by 635 µm. For CA1 spike recordings, microdrives with four to eight channels were constructed in order to independently adjust the depth of recording tetrodes. A single bundle of 30-gauge stainless steel guide tubes spaced the tetrodes by 300 µm. Tetrodes were made of four braided strands of 15 µm NiCr wire (California Fine Wire, USA) and sheathed in polyimide tubing (75 µm ID). Each actuator of the microdrive consisted of a 12.7 mm brass 00-90 hexagonal head machine screw (JI Morris, USA) coupled to a 23-gauge stainless steel drive tube. Tetrodes were threaded through the 30-gauge guide tubes and 23-gauge drive tubes, and individual wires were pinned to a 36 channel electronic interface board (EIB-36N, Neuralynx, USA). Prior to surgery, tetrode tips were precision cut and gold plated with a gold potassium cyanide solution to improve the recording of biopotentials by reducing electrical impedance and tissue reactivity.

### Jugular vein catheterization surgery

Rats were anesthetized with isoflurane (5% induction, 2% maintenance) and an incision made in the upper right quadrant of the thorax to expose the right jugular vein. An indwelling silastic catheter (Dow Corning Corp, USA) was inserted into the jugular vein, and the distal end was run subcutaneously to a port on the dorsal surface of the rat between the scapulae. In the case of rats undergoing electrophysiology recordings, the port was integrated into the headcap.

### Intracranial cannulation

Rats were anesthetized with isoflurane (5% induction, 2% maintenance) and skin excised to expose the skull. Bilateral guide cannulae (26 gauge, 5 mm spacing, Plastics One, USA) were inserted into the brain (AP −3.5 mm bregma, ML ±2.5 mm midline, VD −1.4 mm from dura). Guide cannulae were fixed to the skull with skull screws and dental cement. A microinjector (33 gauge) with a 1 mm extension was utilized to deliver drug.

### Evoked potential electrophysiology surgery

Following jugular vein catheterization, anesthetized rats were placed in a stereotaxic frame, and a 3 by 2.5 mm cranial window (−1.75 to 4.75 mm AP, 47 0.5 to 3.0 mm ML) was drilled over the right dorsal hippocampus. Dura was excised to allow implant of the microwire array in the brain. Lateral to the implanted probe, two individually moveable stimulation electrodes (bipolar 50 µm stainless steel, AM systems) were implanted into the stratum radiatum and stratum oriens, respectively, using standard electrophysiological responses to confirm placement. Electrodes were fixed in place to the skull by dental cement and implanted skull screws, which also served as ground and reference points.

### CA1 Pyramidal spiking electrophysiology surgery

Following jugular vein catheterization, anesthetized rats were placed in a stereotaxic frame, and a 2.0 by 2.0 mm cranial window (−2.3 to 4.3 mm AP, 1.0 to 3.0 mm ML) was drilled over the right dorsal hippocampus. Dura was excised to allow placement of the tip of the microdrive bundle above the cortex overlying the hippocampus. The microdrive was fixed to the skull by dental cement and implanted skull screws, which also served as ground and reference points. In order to record from populations of hippocampal neurons, tetrodes were lowered slowly through the overlying cortex over several weeks of daily sessions while filtered LFP activity (1–475 Hz) and high frequency unit activity (600–6000 Hz) were monitored. The hippocampal pyramidal layer was identified by the characteristic appearance of ripple activity (150–200 Hz) occurring irregularly during immobility, grooming, and sleep. Tetrodes were slowly lowered further until unit activity was observed during ripples, and subsequent tuning took place to maximize stable, separable units.

### Evoked field potential recordings

Rats were acclimated to a recording chamber (40 × 40 × 60 cm) for several days after recovery from surgery. The baseline recording chamber was composed of four uniform dark walls with a removable blue corrugated plastic floor. A single white vertical strip polarized the box for orientation. Extramaze cues were available on all four walls in the recording room. Baseline evoked responses were then tested from 20 to 200 µA stimulation intensity (0.2 ms biphasic stimulation), and a stimulation magnitude evoking 50% of the maximal response was used for the remaining recordings. Baseline recordings were made for a minimum of three days (alternating oriens and radiatum test stimulations, 0.016 Hz per site), and the day prior to testing baseline responses were recorded for 1 h, with brief handling at 30 min. On the test day, drugs (saline, GluA2_3Y_/Scrambled 2.25 µmol/kg) were administered 45 min prior to recording. Rats were randomly assigned to GluA2_3Y_/Scrambled groups. After 30 min in the recording chamber, rats were transferred to a novel environment (60 × 60 × 60 cm polarized box). The novel environment was composed of three black walls and one white wall, with a removable black painted corrugated plastic floor. It was positioned adjacent to the baseline recording box in the same room. Extramaze cues within the room were unchanged. Rats were recorded for 30 min in the novel environment, with test stimulation continuing as before, and then transferred back to the baseline recording chamber for 30 min. On the following day, rats were re-exposed to the novel environment using the same testing protocol as above, with the exception that no drugs were administered prior to testing. Locomotor activity was tracked in 4 of 5 rats treated with Scrambled peptide and 4 of 5 rats treated with GluA2_3Y_ peptide via overhead camera using commercial behavioral tracking software (Ethovision 3.1, Noldus, USA).

### CA1 Place field recording

For place field recordings in a box environment, rats were food restricted and trained to forage for sucrose pellets in a recording chamber (40 × 40 × 60 cm). Baseline recordings were collected during 15-min foraging sessions to confirm the appearance of place specific firing. On the test day, rats received IV infusions (GluA2_3Y_/Scrambled 2.25 µmol/kg in 1 ml/kg saline) 45 min prior to recording. Rats first ran a 15-min foraging session in the familiar configuration, then were removed and placed in a novel environment (60 × 60 × 60 cm polarized box) to run a 30-min foraging session. Rats were then returned to the baseline recording chamber for an 15-min session.

For place field recordings in a linear maze, rats were food restricted and trained to shuttle for sucrose pellets delivered to both ends of a linear maze consisting of four 40 cm segments (width 12 cm, wall height 10 cm) linked by turns of 45°, 90°, and 135°. Rats were trained until a minimum of 8 laps were completed within an 8-min session. On the first exposure day, rats received IV infusions (GluA2_3Y_/Scrambled 2.25 µmol/kg in 1 ml/kg saline) 45 min prior to recording. Rats were randomly assigned to GluA2_3Y_/Scrambled groups. Rats first ran an 8-min shuttling session in the familiar maze configuration, then were removed and placed in a novel configuration to run a 16-min shuttling session. Rats were then returned to the baseline configuration for an 8-min recording session. On the subsequent re-exposure day, rats ran an 8-min shuttling session in the familiar maze configuration, then were removed and placed in the same, previously novel configuration to run a 16-min shuttling session. Rats were then returned to the baseline configuration for an 8-min recording session. Multiple configurations were possible by adjusting the position of the maze in space and the sequence and direction of turns from one end of the maze to the other. Five of seven rats underwent both treatment conditions, with exposure order randomized, while the two remaining animals underwent only one condition (GluA2_3Y_/Scrambled, respectively).

### Inhibitory avoidance

The inhibitory avoidance apparatus consisted of a light and dark chamber separated by a removable door. Each chamber was 35 × 30 × 35 cm, and the stainless steel flooring of the dark compartment was connected to a programmable scrambled shock generator (Colbourne Instruments, USA for systemic experiments, Harvard Apparatus for local injection experiments). Inhibitory avoidance was assessed over a three-day protocol designed to isolate the novel exposure to the recording chamber from the chamber-shock association learning^71,72^. On day one, rats received a contextual exposure to the chamber, in which they were placed in the light side of the box with the door open and allowed to freely explore both sides of box for 8 min. Prior to exploration, rats received bolus IV injection of saline, Scrambled peptide, or GluA2_3Y_ peptide (GluA2_3Y_/Scrambled 2.25 µmol/kg) for systemic experiments, or bilateral microinjection of GluA2_3Y_/Scrambled (45 pmol/µl, 2.5 µl/hemisphere) for local injection experiments 60 min prior to chamber exploration. On day 2, 24 h after contextual exposure, rats were placed directly in the dark chamber with the door closed, and received two brief footshocks 2 s after placement in the chamber (0.4 mA footshock, 0.5 s duration for systemic experiments, 0.55 mA footshock, 0.5 s duration for local injection experiments). Five seconds after termination of the footshock rats were removed from the chamber and returned to their homecage. No drugs were administered in the training session. On day 3, 24 h after training, rats were placed in the light compartment facing the far wall, and after 5 s the door to the dark chamber was opened. Latency to cross entirely into the dark compartment (all four paws within the compartment) was taken as a measure of inhibitory avoidance learning. A control group of rats was administered saline on day one and trained as above, with the exception that no contextual exposure was performed on day one. Drug administration (GluA2_3Y_/Scrambled) or exposure condition were pseudorandomly assigned, with cagemates receiving opposing treatments. Testing on the third day was conducted blind to treatment condition.

### Data acquisition and analysis

Cell spiking, local, and evoked field potentials were recorded using a 32-channel electrophysiology recording system with integrated headstage preamplifier (Digital Lynx 32, HS-36, Neuralynx, AZ). A native sampling rate of 32 kHz was broad-band filtered between 0.1 and 1000 Hz and downsampled to 6.4 kHz for analysis of field potentials. Stimulation (0.2 ms biphasic pulse) was delivered via AM 2100 stimulus isolators, controlled via computer generated TTL pulses. Data were analyzed offline using custom MATLAB analysis code. The amplitude of evoked field potentials was taken as a measure of synaptic strength. As evoked potentials within a recording tract were highly correlated and likely reflected the same population of evoked synapses, only the channel within the tract with the highest magnitude potential was used. This also avoided the analysis of complex waveforms recorded from channels intermediate between stratum oriens and stratum radiatum.

Analysis of standard deviation was adapted from previous work examining hippocampal fEPSPs recorded from multiple electrodes simultaneously^27^. For each channel, a standard deviation threshold was calculated from responses evoked in the baseline period. Responses exceeding this threshold in either direction were counted for each stimulation, resulting in a proportion of above and below threshold channels for each time point for each animal. Proportions were averaged across time for each epoch for analysis.

For spike recordings, positional information was recorded by tracking a colored LED mounted on the headstage preamplifier with an overhead camera (Neuralynx, AZ). Spike sorting was conducted offline using manual spike sorting software (Offline Sorter, Plexon). Putative spikes were preprocessed to remove noise and clusters were identified using 3d projections of spike characteristics. Only well-isolated clusters were selected for analysis. Entire recording sessions, whether containing a single or multiple environmental exposures were sorted collapsed over time. Sorted spike data and LFPs were analyzed using an open source MATLAB toolkit (FMAtoolbox, http://fmatoolbox.sourceforge.net/) and custom MATLAB analysis code. For each recording session, positional information was first filtered to remove target points occurring outside the arena. Tracking jitters were removed by setting a threshold movement speed. Missing points were interpolated from nearby samples and all position samples were smoothed over a ~2 s Gaussian window. Spikes were velocity filtered to remove firing during immobility, defined as movement below 2.5 cm/s over a smoothed 5-second window. For linear track data, two dimensional position samples were linearized to one dimension by collapsing along a single axis defined by a set of vertices corresponding to the end points and three interior corners of the linear maze. Laps were then identified using local extrema on a 25 s smoothed plot of the linearized data. Incomplete laps were removed and subsequent analysis separated inward and outward going laps. Spatial firing rate maps were generated for each isolated unit using a grid of 50 × 50 bins for 2 dimensional box data, while 1 dimensional linearized data used 50 bins.

Cells were identified across days based on previous methods^41,42^. For each cell on each day, the average waveform was calculated, and highly correlated waveforms (*r* > 0.97, taken as a 128 × 1 array of voltage samples, with 32 samples for each tetrode sequentially) recorded from the same tetrode across days in the same rat were taken as a training set of putative matched pairs. Two measures of waveform shape similarity (tolias distance) were calculated for each pair of cells from the same rat, with waveforms expressed as a 32 × 4 array of voltage samples, each column representing each channel of the tetrode. The first (D1) was a measure of shape similarity of waveforms normalized to minimize the sum of squares difference between the two waveforms on each channel. These scaling factors were used to calculate a normalized Euclidean distance between the waveforms on each channel, summing across channels to produce a single D1 value. A separate D2 value, meant to capture both differences between waveform sizes overall and differences between the scaling factor on each channel was also calculated. Plotted on two dimensions, the D1 and D2 values are both small for matching cell pairs, and larger for non-matching pairs. Using the training set, a Bayesian classifier was used to classify all cell pairs as matching or non-matching. A small minority of cells were positively classified to multiple cell on other days, in this case a linearized tolias distance was used to select only the most closely matched cell pair. Firing rate maps for pairs of cells were correlated as two linear arrays using Pearson’s product-moment correlation. The second exposure to the familiar environment in each session was used to compare familiar environmental representations across days.

### Reporting summary

Further information on research design is available in the Nature Research Reporting Summary linked to this article.

## Supplementary information

Supplementary Information

Reporting Summary

## Data Availability

All data supporting the findings of this study are provided within the paper and its Supplementary information. All additional information will be made available upon reasonable request to the authors. Source data are provided with this paper.

## Source data

Source Data

## Code availability

The code that support the findings of this study are available at https://github.com/donovanashby/ltd-pc and used under GNU General Public License (GPL).
