# Supplementary material for: LTD is involved in the formation and maintenance of rat hippocampal CA1 place-cell fields
Source: Nat Commun. 2021 Jan 4;12:100. doi: 10.1038/s41467-020-20317-7 (PMC7782827; doi:10.1038/s41467-020-20317-7)
Supplement: Supplementary file 1 — Supplementary Information [file 41467_2020_20317_MOESM1_ESM.pdf]

LTD is involved in the formation and maintenance of rat hippocampal CA1 place-cell fields

Ashby et al., Supplementary Figures 1-7.

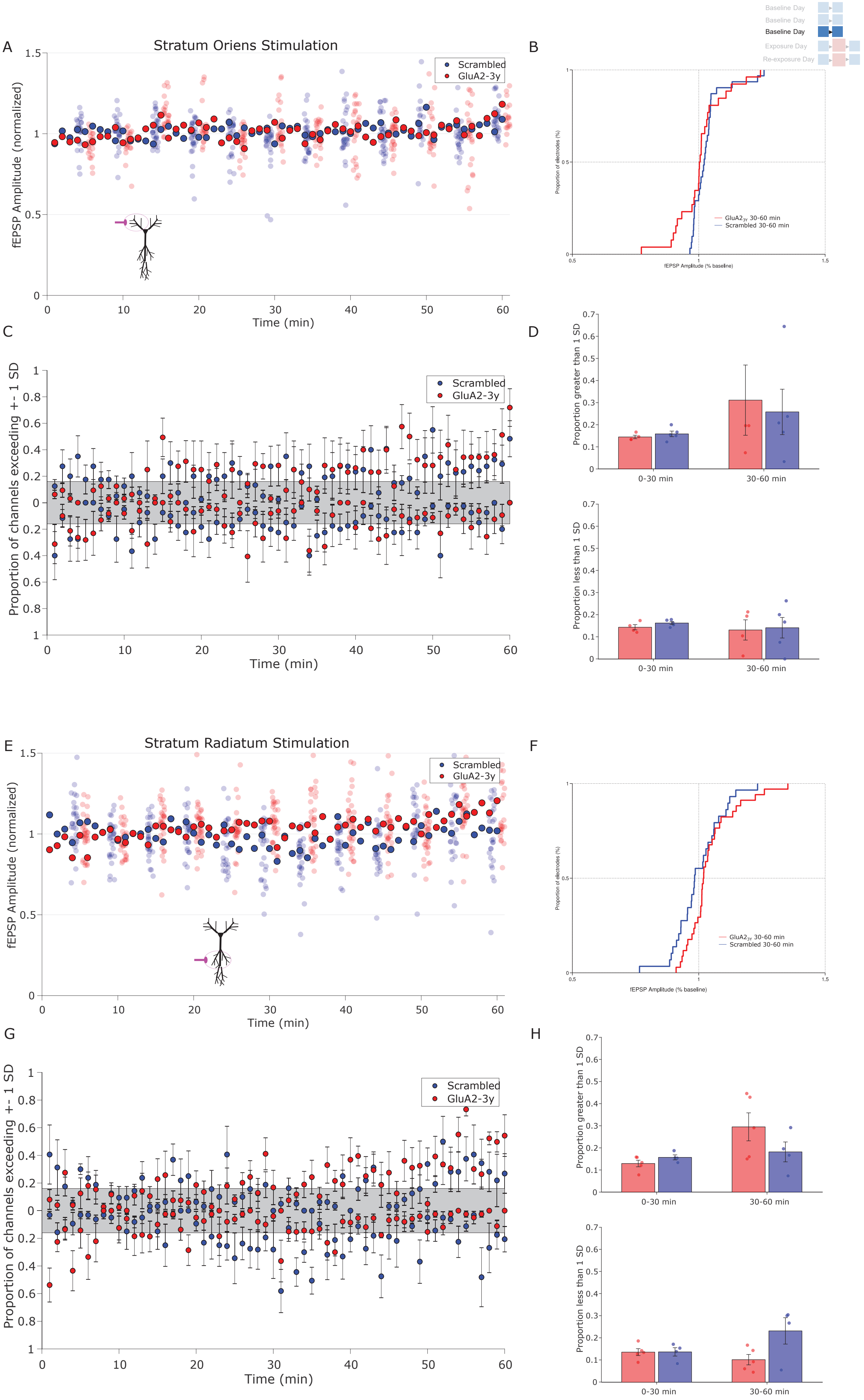

Baseline Day
Baseline Day
Baseline Day
Exposure Day
Re-exposure Day
A
Stratum Oriens Stimulation
fEPSP Amplitude (normalized)
Time (min)
Scrambled
GluA2-3y
B
Proportion of electrodes (%)
fEPSP Amplitude (% baseline)
GluA2$_{3y}$ 30-60 min
Scrambled 30-60 min
C
Proportion of channels exceeding +- 1 SD
Time (min)
Scrambled
GluA2-3y
D
Proportion greater than 1 SD
Proportion less than 1 SD
0-30 min
30-60 min
E
Stratum Radiatum Stimulation
fEPSP Amplitude (normalized)
Time (min)
Scrambled
GluA2-3y
F
Proportion of electrodes (%)
fEPSP Amplitude (% baseline)
GluA2$_{3y}$ 30-60 min
Scrambled 30-60 min
G
Proportion of channels exceeding +- 1 SD
Time (min)
Scrambled
GluA2-3y
H
Proportion greater than 1 SD
Proportion less than 1 SD
0-30 min
30-60 min

Supplementary Figure 1. Final baseline day evoked field potentials are stable. (A) Hippocampal CA1 fEPSPs evoked from stimulation of the oriens pathway in rats subsequently treated with Scrambled (blue) or GluA23y peptide (red). Solid marker indicates the cross-channel average, while each channel is plotted every 5 stimulations and offset for clarity. (B) Distribution of fEPSP change in the second 30-minute epoch relative to the first. No difference in distribution was observed (n=31 channels from scrambled treated rats, n=26 channels from GluA23y treated rats). (C) Per-animal proportion of channels exceeding 1 standard deviation of the channel's baseline variance at each stimulation. Shaded area represents the proportion of channels expected to be above or below 1 standard deviation based on a normal distribution (n=4 GluA23Y, n=5 Scrambled). (D) Proportion of channels exceeding 1 standard deviation above (top graph) or below (bottom graph) baseline, in 30-minute bins. No significant difference in variance proportion was observed (n=4 GluA23Y, n=5 Scrambled). (E) fEPSPs evoked from stimulation of the radiatum pathway in rats subsequently treated with Scrambled (blue, n=29) or GluA23y (red, n=34). Solid marker indicates the cross-channel average, while each channel is plotted every 5 stimulations and offset for clarity. (F) Distribution of evoked fEPSP change in the second 30-minute epoch relative to the first. No difference in distributions was observed. (G) Per-animal proportion of channels exceeding 1 standard deviation of the channel's baseline variance at each stimulation (n=5 GluA23Y, n=4 Scrambled). (H) Proportion of channels exceeding 1 standard deviation above (top graph) or below (bottom graph) baseline, in 30-minute bins. A non-specific increase in the proportion of channels higher than baseline variance was observed ($F_{Epoch}(1,7)=7.66$, p=.028). An interaction was observed in the proportion of channels lower than baseline variance ($F_{Epoch*Group}(1,7)=9.23$, p=.019) but follow-up pairwise comparisons (t-test, two tailed) indicated no significant difference between treatment groups in either epoch (ps>.05, n=5 GluA23Y, n=4 Scrambled). Animals were briefly handled at 30 minutes. Error bars represent +-SEM.

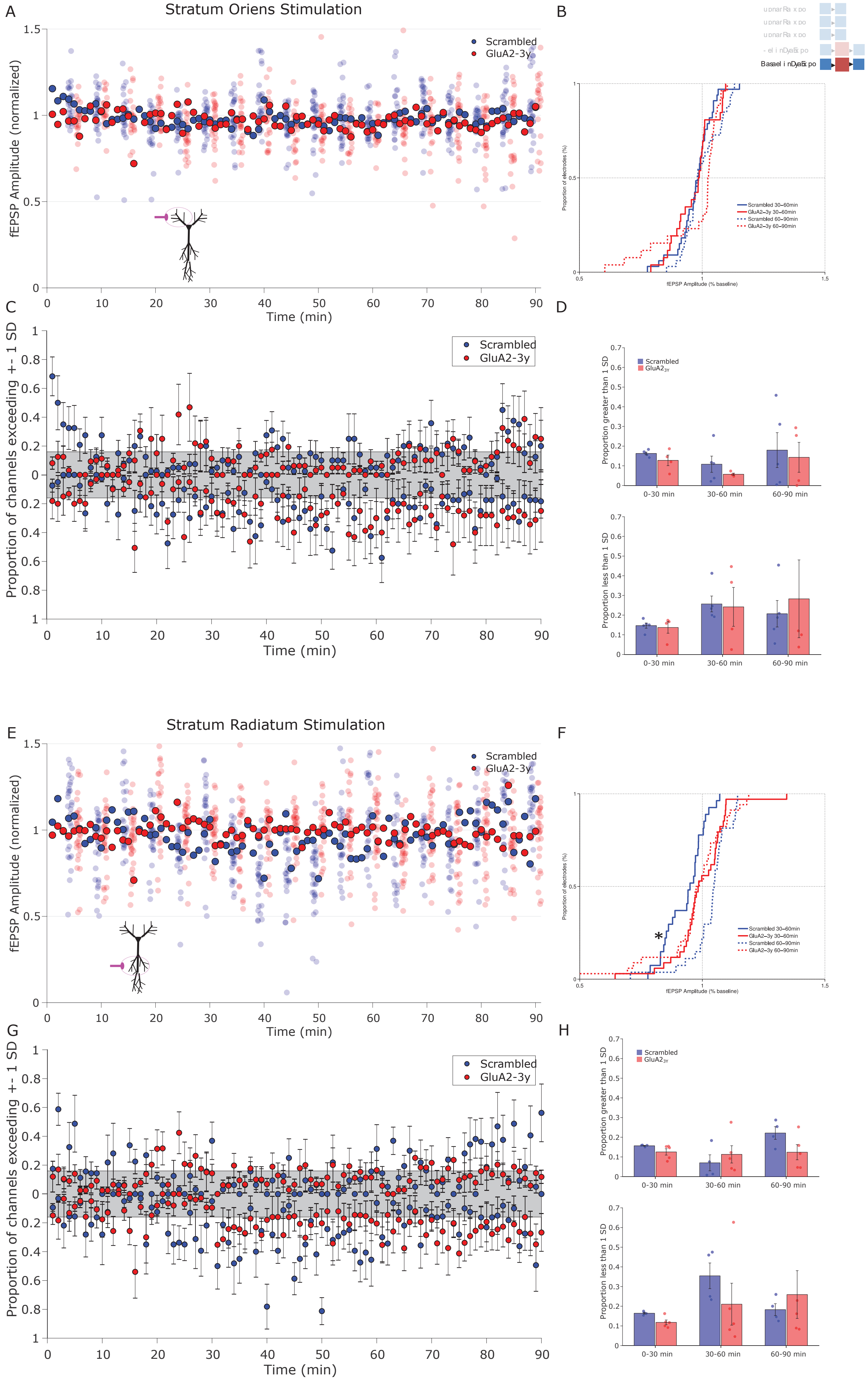
A
Stratum Oriens Stimulation
Scrambled
GluA2-3y
fEPSP Amplitude (normalized)
Time (min)
B
Proportion of electrodes (%)
fEPSP Amplitude (% baseline)
Scrambled 30–60min
GluA2–3y 30–60min
Scrambled 60–90min
GluA2–3y 60–90min
C
Proportion of channels exceeding +- 1 SD
Time (min)
D
Proportion greater than 1 SD
Proportion less than 1 SD
0-30 min
30-60 min
60-90 min
E
Stratum Radiatum Stimulation
F
*
G
H

Supplementary Figure 2. Re-exposure day evoked field potentials do not show *de novo* LTD. (A) Hippocampal CA1 fEPSPs evoked from stimulation of the oriens pathway in rats previously treated with Scrambled (blue, n=31) or GluA23y peptide (red, n=26). Solid marker indicates the cross-channel average, while each channel is plotted every 5 stimulations and offset for clarity. A 30-minute recording session in the familiar baseline environment was followed by a 30-minute re-exploration of the novel environment, first explored on the previous day. This was followed by a return to the familiar environment for 30-minutes. (B) Distribution of fEPSP change during (30-60 min) and after (60-90 min) re-exploration of the novel environment relative to the first 30 minutes. No difference in distribution was observed. (C) Per-animal proportion of channels exceeding 1 standard deviation of the channel's baseline variance at each stimulation. Shaded area represents the proportion of channels expected to be above or below 1 standard deviation based on a normal distribution (n=4 GluA23Y, n=5 Scrambled). (D) Proportion of channels exceeding 1 standard deviation above (top graph) or below (bottom graph) baseline, in 30-minute bins. No significant difference in variance proportion was observed (n=4 GluA23Y, n=5 Scrambled). (E) fEPSPs evoked from stimulation of the radiatum pathway in rats previously treated with Scrambled (blue, n=29) or GluA23y (red, n=34). Solid marker indicates the cross-channel average, while each channel is plotted every 5 stimulations and offset for clarity. (F) Distribution of evoked fEPSP change during (30-60 min) and after (60-90 min) re-exploration of the novel environment relative to the first 30 minutes. A transient decrease in the distribution of fEPSPs from Scrambled rats relative to GluA23Y (ks=0.59, p<.001) was reversed to a marginal increase (ks=.032, p=.06) after novel exploration (n=5 GluA23Y, n=4 Scrambled). (G) Per-animal proportion of channels exceeding 1 standard deviation of the channel's baseline variance at each stimulation. (H) Proportion of channels exceeding 1 standard deviation above (top graph) or below (bottom graph) baseline, in 30-minute bins. An effect of epoch ($F_{Epoch}(2,7)=4.20$, p=.037) and marginal interaction ($F_{Epoch*Group}(2,7)=3.74$, p=.050) in the proportion of channels higher than baseline variance was observed, however pairwise comparisons (t-test, two-tailed) indicated no significant difference between Scrambled and GluA23Y treatment groups (n=5 GluA23Y, n=4 Scrambled). *p<.001, ks test. Error bars represent +-SEM.

A

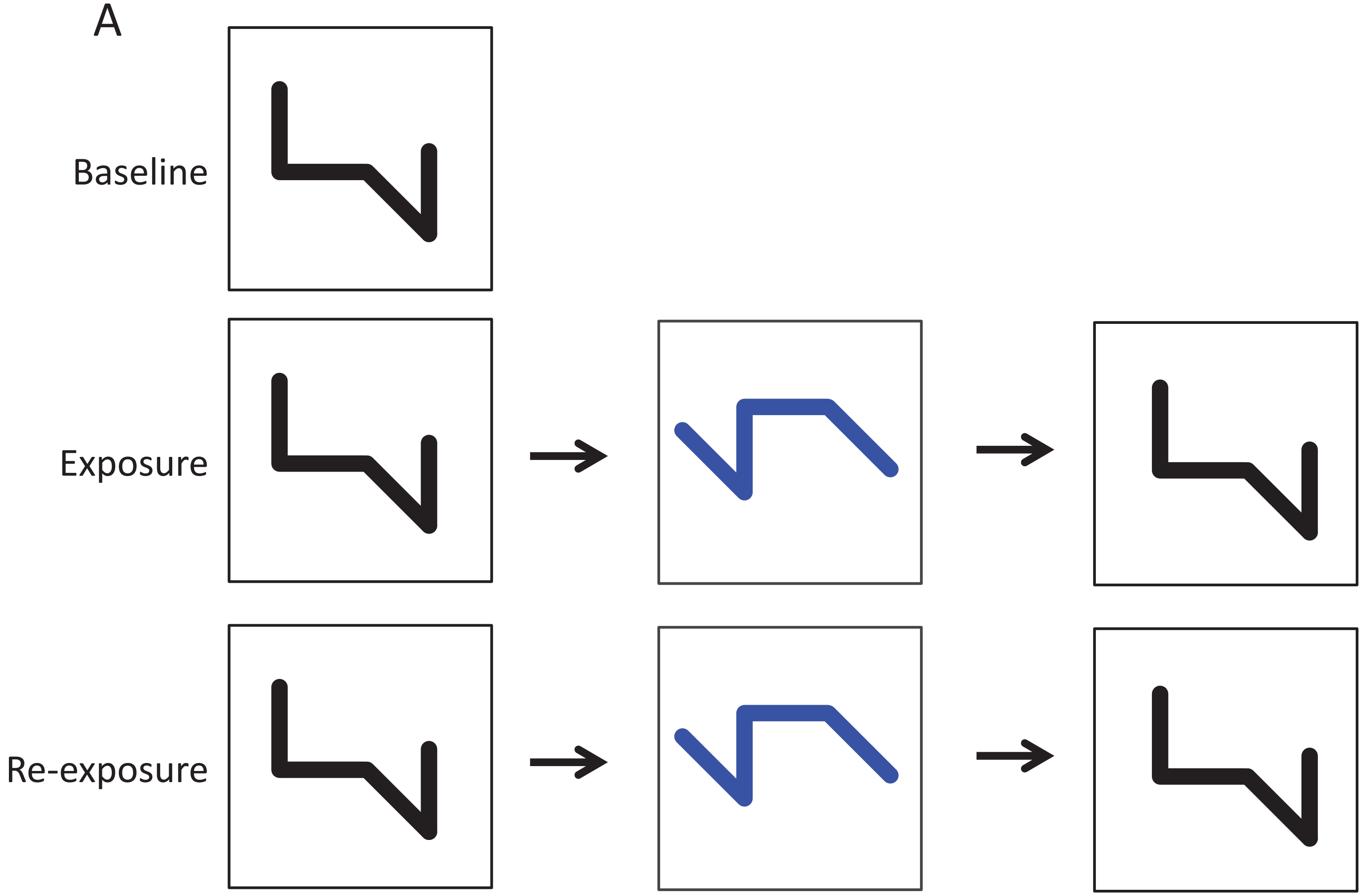

Supplementary Figure 3. Novel reconfigurations of a linear maze. (A). A familiar configuration of four maze sections was recorded on a baseline day. A novel reconfiguration of the maze and external landmarks was presented first on an exposure day, and the same reconfiguration was presented on a re-exposure day. All reconfigurations included both trajectory and allocentric location changes, rather than simple rotations/translations.

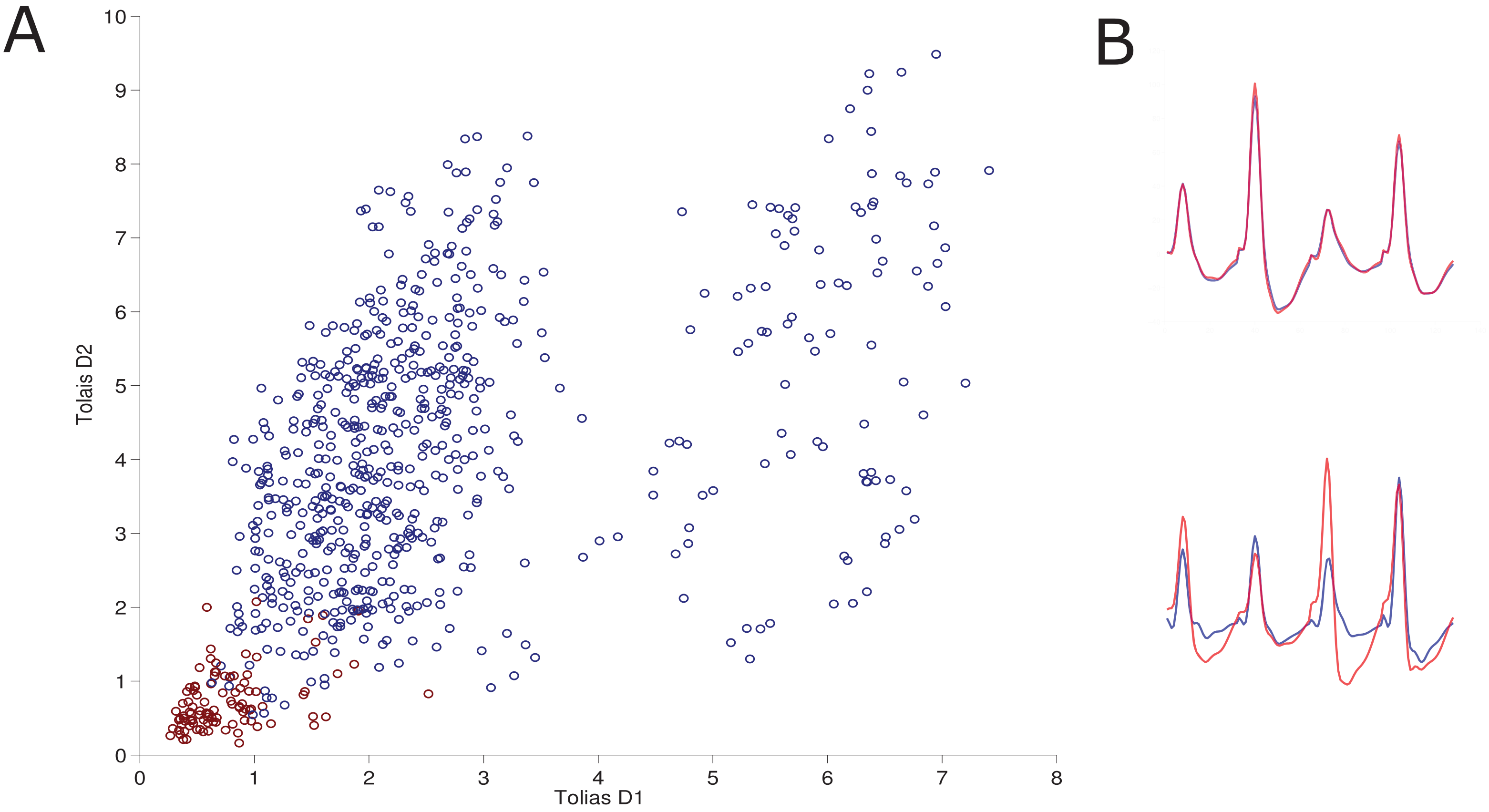

A
B
Tolais D2
Tolias D1
0
1
2
3
4
5
6
7
8
9
10

Supplementary Figure 4. Identification of Cells across Days. (A) Average waveforms for each sorted cell were compared to average waveforms on the subsequent day recorded from the same tetrode. Projection of computed tolias distance value D1 (x-axis) and D2 (y-axis) indicated a subset of highly similar waveforms (red), classified with a 2-dimensional Bayesian classifier trained on a subset of highly correlated waveforms ($p>.97$). (B) Example tetrode waveforms from matched and non-matched pairs of cells. Each peak represents the average waveform recorded on each of four tetrode channels

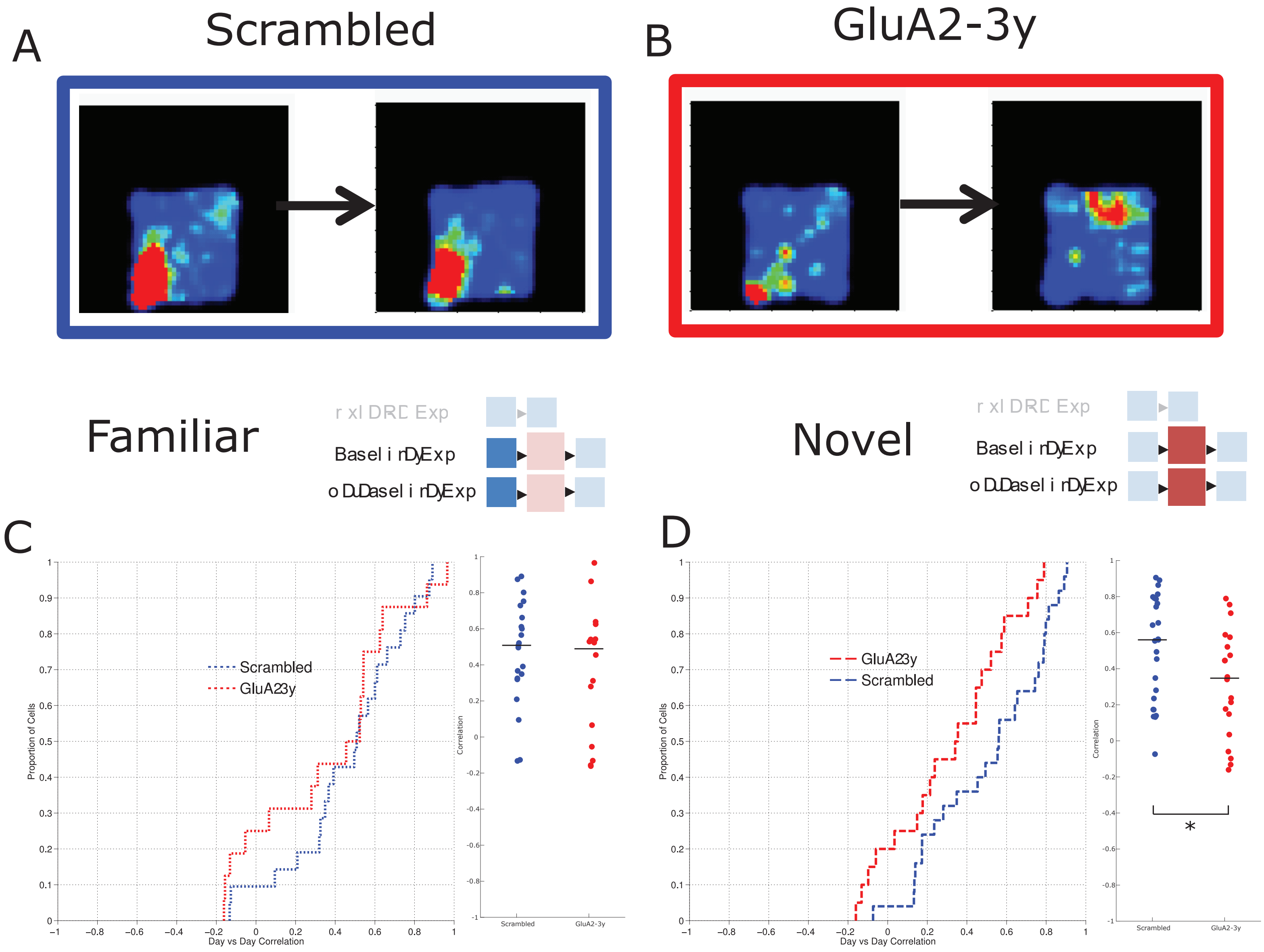
A
Scrambled
B
GluA2-3y
Familiar
Novel
C
D
Scrambled
GluA23y
Proportion of Cells
Day vs Day Correlation
Correlation
GluA2-3y
*

Supplementary Figure 5. LTD blockade disrupts place field maintenance in a novel Square Maze. 218 separable cells were recorded from area CA1 in eight rats over three recording days in which rats explored a novel box environment on the second day. Place field firing was assessed in a highly familiar environment on one baseline day, and GluA23Y (2.25 µmol/kg IV) or a scrambled control was administered prior to recording on the second day, in which a novel environment was presented after a brief exploration of the familiar environment. Following novel environmental exposure, rats were returned to the familiar environment. The same protocol was run on the following day in the absence of any drug administration. Place field stability was measured as a correlation between the firing maps produced in two different sessions from cells identified as matching. A total of n=92 cells from N=8 rats were identifiable across consecutive days in the box environment condition. Cells were analyzed for correlated place activity across days if they showed a place field within the analyzed environment (>1 Hz peak firing rate) in at least one recording session. (A,B) Examples of cells recorded from scramble peptide treated (A) and GluA2-3y peptide treated rats (B), on first exposure to the novel environment and on re-exposure the following day, in a square novel environment. (C) Day over day correlation between exposure day and re-exposure day in the familiar recording chamber. Firing fields for cells recorded from scrambled treated rats (blue) and from cells recorded from GluA2-3y (red) treated rats were similarly correlated in the familiar environment, with relatively conserved field firing location. (D) Day over day correlation between exposure day and re-exposure day in the novel environment. Firing fields for cells recorded from scrambled treated rats (blue) were more highly correlated across days as compared with firing fields from cells recorded from GluA2-3y treated rats (red, U=664, p=.043). As familiar and novel environments were of different sizes, place fields were not directly comparable, so no omnibus comparison was conducted between familiar and novel environments. Distribution were not significantly different between scrambled and GluA23y in either familiar or novel environments (ps>.05, ks-test). * indicates p<.05 Mann-Whitney U.

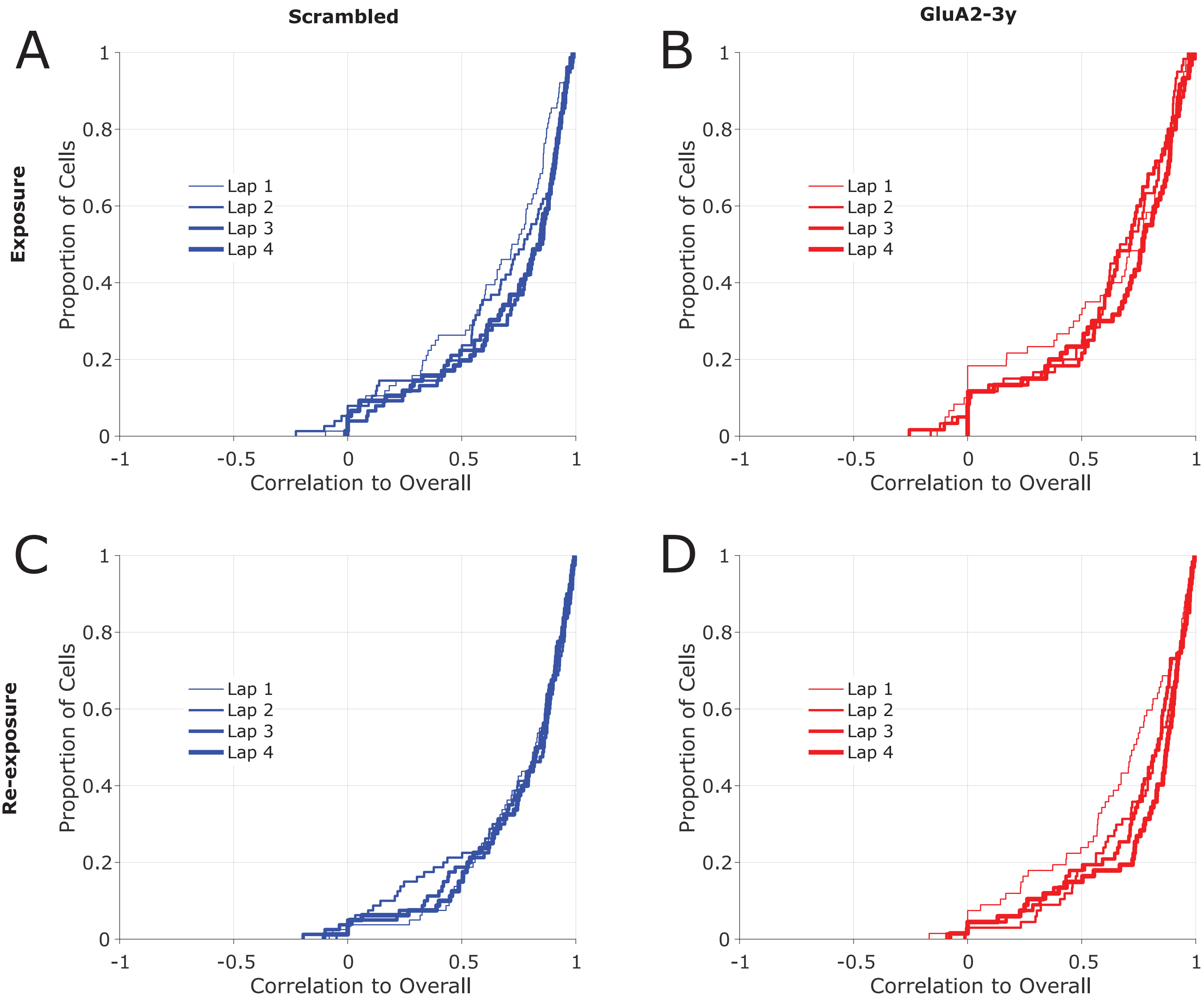
Scrambled
GluA2-3y
Exposure
Re-exposure
A
B
C
D
Proportion of Cells
Correlation to Overall
Lap 1
Lap 2
Lap 3
Lap 4
0
0.2
0.4
0.6
0.8
1
-1
-0.5
0
0.5
1

Supplementary Figure 6. Place fields are stable in early laps of a familiar environment. Early laps on first exposure to the familiar linear maze showed immediately highly correlated place fields in scrambled treated rats on the novelty exposure day. (B) Similarly high correlations were observed in GluA23y treated rats. (C) Re-exploration of the familiar maze immediately after novelty exposure did not affect early field stability in Scrambled treated rats or (D) GluA23y treated rats. All ps>.05, ks-test.

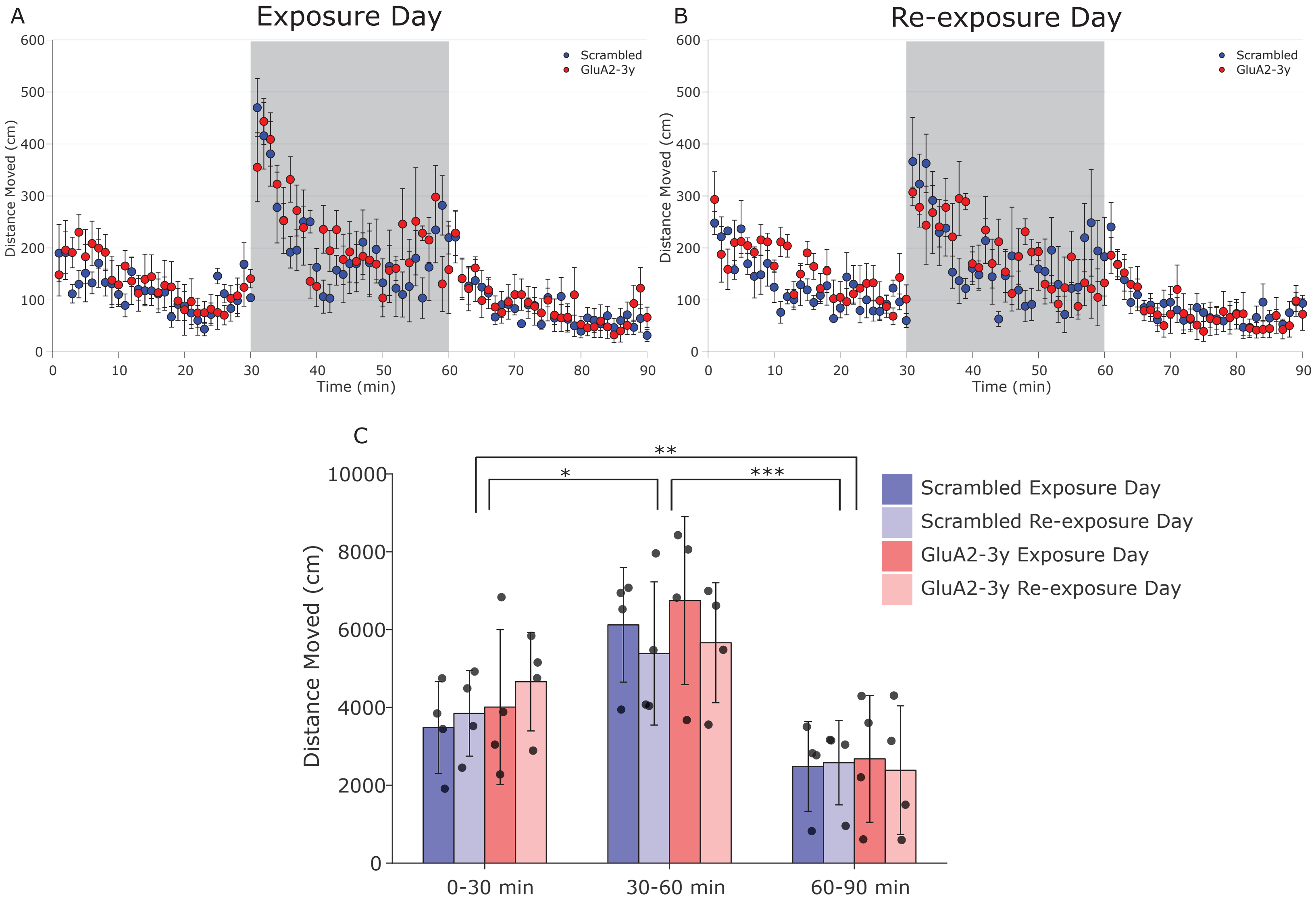
A
Exposure Day
B
Re-exposure Day
Scrambled
GluA2-3y
Distance Moved (cm)
Time (min)
C
Distance Moved (cm)
0-30 min
30-60 min
60-90 min
Scrambled Exposure Day
Scrambled Re-exposure Day
GluA2-3y Exposure Day
GluA2-3y Re-exposure Day
*
**
***

Supplementary Figure 7. Locomotor Activity. (A) Locomotor activity in a 30-minute recording session in the familiar baseline environment, followed by a 30-minute exploration of the novel environment, followed by 30 minutes in the familiar environment. Rats were treated with Scrambled (n=4) or GluA23Y (n-4) peptide. (B) Locomotor activity in a 30-minute recording session in the familiar baseline environment, followed by a 30-minute re-exploration of the novel environment, followed by 30 minutes in the familiar environment. (C) Total distance moved in each epoch was analyzed with a three way mixed model ANOVA. Only a significant effect of epoch ($F_{Epoch}(2,12)=21.313$, $p<.001$) was observed. Follow-up pairwise comparison between epochs (baseline vs exposure p=.016, baseline vs post-novelty p=.009, exposure vs post-novelty p=.001) indicated that locomotor was significantly higher in the novel environment than both epochs in the familiar environment. * p<.05, ** p<.01, ***p<.001. Error bars represent +-SEM.
