# Supplementary material for: LTD is involved in the formation and maintenance of rat hippocampal CA1 place-cell fields
Source: Nat Commun. 2021 Jan 4;12:100. doi: 10.1038/s41467-020-20317-7 (PMC7782827; doi:10.1038/s41467-020-20317-7)
Supplement: Supplementary file 2 — Reporting Summary [file 41467_2020_20317_MOESM2_ESM.pdf]

nature research

Corresponding author(s): Yu Tian Wang
Last updated by author(s): Oct 26, 2020

Reporting Summary

Nature Research wishes to improve the reproducibility of the work that we publish. This form provides structure for consistency and transparency in reporting. For further information on Nature Research policies, see our Editorial Policies and the Editorial Policy Checklist.

Statistics

For all statistical analyses, confirm that the following items are present in the figure legend, table legend, main text, or Methods section.

n/a	Confirmed	
☐	☒	The exact sample size (n) for each experimental group/condition, given as a discrete number and unit of measurement
☒	☐	A statement on whether measurements were taken from distinct samples or whether the same sample was measured repeatedly
☐	☒	The statistical test(s) used AND whether they are one- or two-sided<br>*Only common tests should be described solely by name; describe more complex techniques in the Methods section.*
☒	☐	A description of all covariates tested
☐	☒	A description of any assumptions or corrections, such as tests of normality and adjustment for multiple comparisons
☐	☒	A full description of the statistical parameters including central tendency (e.g. means) or other basic estimates (e.g. regression coefficient) AND variation (e.g. standard deviation) or associated estimates of uncertainty (e.g. confidence intervals)
☐	☒	For null hypothesis testing, the test statistic (e.g. F, t, r) with confidence intervals, effect sizes, degrees of freedom and P value noted<br>*Give P values as exact values whenever suitable.*
☒	☐	For Bayesian analysis, information on the choice of priors and Markov chain Monte Carlo settings
☒	☐	For hierarchical and complex designs, identification of the appropriate level for tests and full reporting of outcomes
☒	☐	Estimates of effect sizes (e.g. Cohen's d, Pearson's r), indicating how they were calculated

Our web collection on statistics for biologists contains articles on many of the points above.

Software and code

Policy information about availability of computer code

Data collection	Cheetah 5.6 (Neuralynx), Ethovision 3.1 (Noldus).
Data analysis	Matlab Import/Export Utilities (Neuralynx), Offline Sorter (Plexon), MATLAB, FMAToolbox, custom code (MATLAB)

For manuscripts utilizing custom algorithms or software that are central to the research but not yet described in published literature, software must be made available to editors and reviewers. We strongly encourage code deposition in a community repository (e.g. GitHub). See the Nature Research guidelines for submitting code & software for further information.

Data

Policy information about availability of data

All manuscripts must include a data availability statement. This statement should provide the following information, where applicable:

- Accession codes, unique identifiers, or web links for publicly available datasets
- A list of figures that have associated raw data
- A description of any restrictions on data availability

The data that support the findings of this study are available from the corresponding author upon reasonable request.

Field-specific reporting

Life sciences study design

All studies must disclose on these points even when the disclosure is negative.

Sample size	Sample sizes based on prior published, peer review work using similar approaches for electrophysiology and behavior: Multi-channel evoked field recordings (Whitlock et al., 2006), Place field recordings with plasticity modifying drugs (Kentros, 1998), Context pre-exposure inhibitory avoidance (Roesler, 1998).
Data exclusions	no data excluded
Replication	The primary LTD effect observed with saline treatment was replicated in a follow-up experiment with a scrambled control peptide (1x independent replication). The effect of LTD blockade on place field maintenance was reproduced in a different maze configuration (1x independent replication). No replication of the effect of LTD blockade on place field formation dynamics was attempted. The behavioral effect of systemic LTD blockade was reproduced with a local injection experiment (1x independent replication). Although these are not strict replications they support the reproducibility of the key findings.
Randomization	Drug administration groups were assigned randomly for evoked field recordings, and counterbalanced randomly for place field recordings. Behavioral groups were pseudorandomly assigned such that cagemates were in opposing groups.
Blinding	Investigators were blinded to group assignments during offline cell sorting, which was the only subjective analysis step. Neural data was acquired and analyzed using automated approaches.

Reporting for specific materials, systems and methods

We require information from authors about some types of materials, experimental systems and methods used in many studies. Here, indicate whether each material, system or method listed is relevant to your study. If you are not sure if a list item applies to your research, read the appropriate section before selecting a response.

Materials & experimental systems

n/a	Involved in the study
☒	☐ Antibodies
☒	☐ Eukaryotic cell lines
☒	☐ Palaeontology and archaeology
☐	☒ Animals and other organisms
☒	☐ Human research participants
☒	☐ Clinical data
☒	☐ Dual use research of concern

Methods

n/a	Involved in the study
☒	☐ ChIP-seq
☒	☐ Flow cytometry
☒	☐ MRI-based neuroimaging

Animals and other organisms

Policy information about studies involving animals; ARRIVE guidelines recommended for reporting animal research

Laboratory animals	Rat, Sprague-Dawley, Male, 3-6 months
Wild animals	study did not involve wild animals
Field-collected samples	study did not involve field collected samples
Ethics oversight	UBC Animal Care Committe (ACC), Canadian Council on Animal Care (CCAC)

Note that full information on the approval of the study protocol must also be provided in the manuscript.
