# Supplementary material for: LTD is involved in the formation and maintenance of rat hippocampal CA1 place-cell fields
Source: Nat Commun. 2021 Jan 4;12:100. doi: 10.1038/s41467-020-20317-7 (PMC7782827; doi:10.1038/s41467-020-20317-7)
Supplement: Supplementary file 3 — Source Data [file 41467_2020_20317_MOESM3_ESM.zip › stats_table.pdf]

Supplementary Table 1. Statistical Tests

Figure 1

Baseline Day, Saline, Oriens vs Radiatum Distribution

	ks	p
Epoch 30-60 min	0.18	0.497

Baseline Day, Saline, Oriens vs Radiatum, Deviation below

	df	F	p
Epoch	1,8	0.11	0.752
Group	1,8	0.23	0.646
Epoch*Group	1,8	0.05	0.821

Baseline Day, Saline, Oriens vs Radiatum, Deviation below

	df	F	p
Epoch	1,8	4.44	0.068
Group	1,8	0.43	0.530
Epoch*Group	1,8	0.36	0.564

Exposure Day, Saline, Oriens vs Radiatum Distribution

	ks	p
Epoch 30-60 min	0.37	***0.008***
Epoch 60-90 min	0.47	***0.000***

Exposure Day, Saline, Oriens vs Radiatum, Deviation below

	df	F	p
Epoch	2,8	16.17	***0.000***
Group	1,8	5.81	***0.042***
Epoch*Group	2,8	5.49	***0.015***

Exposure Day, Saline, Oriens vs Radiatum, Deviation above

	df	F	p
Epoch	2,8	1.47	0.259
Group	1,8	3.69	0.091
Epoch*Group	2,8	4.08	***0.037***

Figure 2

Exposure Day, Radiatum, Scrambled vs GluA23y Distribution

	ks	p
Epoch 30-60 min	0.28	0.184
Epoch 60-90 min	0.49	***0.001***

Exposure Day, Radiatum, Scrambled vs GluA23y, Deviation Below

	df	F	p
Epoch	2,7	3.21	0.071
Group	1,7	8.11	***0.025***
Epoch*Group	2,7	3.60	***0.055***

Exposure Day, Radiatum, Scrambled vs GluA23y, Deviation Above

	df	F	p
Epoch	2,7	1.42	0.275
Group	1,7	1.96	0.204
Epoch*Group	2,7	1.96	0.178

Exposure Day, Oriens, Scrambled vs GluA23y Distribution

	ks	p
Epoch 30-60 min	0.26	0.216
Epoch 60-90 min	0.32	0.083

Exposure Day, Oriens, Scrambled vs GluA23y, Deviation Below

	df	F	p
Epoch	2,7	2.23	0.144
Group	1,7	0.43	0.531
Epoch*Group	2,7	0.28	0.759

Exposure Day, Oriens, Scrambled vs GluA23y, Deviation Above

	df	F	p
Epoch	2,7	1.22	0.324
Group	1,7	0.15	0.709
Epoch*Group	2,7	0.00	0.998

Figure 3

Linear Maze, All, Scrambled vs GluA23y, Distribution

Maze	Day	ks	p
Familiar	1 vs 2	0.19	0.448
Familiar	2 vs 3	0.21	0.212
Novel	2 vs 3	0.31	***0.003***

Linear Maze, Rate Stable, Scrambled vs GluA23y, Distribution

Maze	Day	ks	p
Familiar	1 vs 2	0.19	0.448
Familiar	2 vs 3	0.22	0.472
Novel	2 vs 3	0.40	***0.013***

Linear Maze, Scrambled vs GluA23y, Familiar vs Novel, Day 2 vs 3

	df	chi2	p
All	3,229	16.07	***0.001***
Rate-Stable	3,112	14.02	***0.003***

Linear Maze, All, Scrambled vs GluA23y, Day 2 vs 3, Followup

		U	p
Familiar	2 vs 3	3150	0.188
Novel	2 vs 3	5617	***0.010***

Linear Maze, Rate-Stable, Scrambled vs GluA23y, Day 2 vs 3, Followup

		U	p
Familiar	2 vs 3	872	0.594
Novel	2 vs 3	1266	***0.008***

Figure 4

Linear Maze, Exposure Day, Distribution vs 4th Lap

Treatment	Lap	ks	p
Scrambled	1 vs 4	0.53	***0.000***

Scrambled	2 vs 4	0.29	***0.002***
Scrambled	3 vs 4	0.22	***0.015***
GluA23y	1 vs 4	0.38	***0.000***
GluA23y	2 vs 4	0.21	0.070
GluA23y	3 vs 4	0.15	0.343

Figure 5

Inhibitory Avoidance, IV Administration

	df	t	p
PE vs NPE	14	2.27	**0.039**
Scr vs GluA2	27	2.25	**0.033**

Inhibitory Avoidance, IC Administration

	df	t	p
Scr vs GluA2	22	2.71	**0.013**

Sup Figure 1

Baseline Day, Radiatum, Scrambled vs GluA23y Distribution

	ks	p
Epoch 30-60 min	0.29	0.125

Baseline Day, Oriens, Scrambled vs GluA23y Distribution

	ks	p
Epoch 30-60 min	0.23	0.370

Baseline Day, Radiatum, Scrambled vs GluA23y, Deviation Below

	df	F	p
Epoch	1,7	1.21	0.307
Group	1,7	2.74	0.142
Epoch*Group	1,7	9.23	***0.019***

Baseline Day, Radiatum, Scrambled vs GluA23y, Deviation Above

	df	F	p
Epoch	1,7	7.66	***0.028***
Group	1,7	0.85	0.386
Epoch*Group	1,7	3.54	0.102

Baseline Day, Oriens, Scrambled vs GluA23y, Deviation Below

	df	F	p
Epoch	1,7	0.23	0.646
Group	1,7	0.22	0.652
Epoch*Group	1,7	0.02	0.904

Baseline Day, Oriens, Scrambled vs GluA23y, Deviation Above

	df	F	p
Epoch	1,7	1.80	0.221
Group	1,7	0.05	0.824
Epoch*Group	1,7	0.12	0.740

Sup Figure 2

Re-exposure Day, Radiatum, Scrambled vs GluA23y Distribution

	ks	p
Epoch 30-60 min	0.59	**0.000**
Epoch 60-90 min	0.32	0.062

Re-exposure Day, Radiatum, Scrambled vs GluA23y, Deviation Below

	df	F	p
Epoch	2,7	2.76	0.098
Group	1,7	0.18	0.683
Epoch*Group	2,7	1.76	0.208

Re-exposure Day, Radiatum, Scrambled vs GluA23y, Deviation Above

	df	F	p
Epoch	2,7	4.20	***0.037***
Group	1,7	0.59	0.468
Epoch*Group	2,7	3.74	***0.050***

Re-exposure Day, Oriens, Scrambled vs GluA23y Distribution

	ks	p
Epoch 30-60 min	0.32	0.062
Epoch 60-90 min	0.28	0.169

Re-exposure Day, Oriens, Scrambled vs GluA23y, Deviation Below

	df	F	p
Epoch	2,7	1.56	0.245
Group	1,7	0.03	0.864
Epoch*Group	2,7	0.28	0.760

Re-exposure Day, Oriens, Scrambled vs GluA23y, Deviation Above

	df	F	p
Epoch	2,7	1.61	0.235
Group	1,7	0.56	0.477
Epoch*Group	2,7	0.02	0.982

Sup Figure 5

Box Maze, All, Scrambled vs GluA23y

		U	p
Familiar	1 vs 2	287	0.751
Familiar	2 vs 3	427	0.399
Novel	2 vs 3	664	***0.043***

Sup Figure 7

Locomotor Activity, Evoked Potential Electrophysiology

	df	F	p
Day	1,6	0.30	0.601
Epoch	2,12	21.31	***0.000***
Group	2,7	0.23	0.651
Day*Epoch	2,12	2.90	0.094

Day*Group	1,6	0.06	0.809
Epoch*Grou	2,12	0.21	0.817
3-way	2,12	0.21	0.811
